# Interpretation of Pharmacometabolomics Results: Fingerprint of Drug Exposure or Confounder Effects? Insights from a Urinary Metabolomics Study with Voriconazole in Healthy Participants

**DOI:** 10.3390/ijms27104468

**Published:** 2026-05-16

**Authors:** Kristine Chobanyan-Jürgens, Amin Muhareb, Moritz Niesert, Camilo Scherkl, Andreas D. Meid, Claire Cannet, Dora Pituk, Georg F. Hoffmann, Julia C. Stingl, Andreas Ziegler, Antje Blank

**Affiliations:** 1 Heidelberg University, Medical Faculty Heidelberg, Heidelberg University Hospital, Internal Medicine IX–Department of Clinical Pharmacology and Pharmacoepidemiology, Im Neuenheimer Feld 410, 69120 Heidelberg, Germany; amin.muhareb@ukbonn.de (A.M.); moritz.niesert@med.uni-heidelberg.de (M.N.); camilo.scherkl@med.uni-heidelberg.de (C.S.); andreas.meid@med.uni-heidelberg.de (A.D.M.); juliacarolin.stingl@med.uni-heidelberg.de (J.C.S.); andreas.ziegler@med.uni-heidelberg.de (A.Z.); antje.blank@med.uni-heidelberg.de (A.B.); 2 Heidelberg University, Medical Faculty Heidelberg, Heidelberg University Hospital, Center for Pediatrics and Adolescent Medicine, Department of Pediatrics I, Im Neuenheimer Feld 430, 69120 Heidelberg, Germany; georg.hoffmann@med.uni-heidelberg.de; 3 Heidelberg University, Medical Faculty Heidelberg, Heidelberg University Hospital, Pediatric Clinical-Pharmacological Trial Center (paedKliPS), Im Neuenheimer Feld 430, 69120 Heidelberg, Germany; 4 Bruker BioSpin GmbH & Co. KG, 76275 Ettlingen, Germany; claire.cannet@bruker.com (C.C.); dora.pituk-extern@bruker.com (D.P.)

**Keywords:** clinical trial, CYP3A4 inhibition, fasting, urinary pharmacometabolomics, proton nuclear magnetic resonance spectroscopy, sodium bicarbonate, voriconazole

## Abstract

Interpretation of pharmacometabolomics results, aiming particularly at biomarker (sets) discovery for drug exposure, remains a major challenge. The metabotyping of drug exposure depends on resolution of specific metabolomics techniques and comprises individual metabolic phenotypes (“metabotypes”), disease-, drug- and microbiome-specific patterns, as well as conditional metabolic states (e. g. fasting). In this clinical trial with 16 healthy participants, an exploratory objective was to evaluate the untargeted urinary metabolomics of voriconazole, administered in four single doses, using proton nuclear magnetic resonance (^1^H-NMR) spectroscopy. Voriconazole is a second-generation triazole and a potent inhibitor of drug-metabolizing enzymes such as cytochrome P450 (CYP) isozymes CYP3A4 and CYP2C19. Therefore, identification of metabolites reflecting acute CYP3A4 inhibition was of particular interest. On two treatment days without and with voriconazole (with background microdosed midazolam and omeprazole administration for CYP3A4 and CYP2C19 phenotyping, respectively), spot urine was collected after overnight fasting (predose) and 4 h later (postdose fasting). In the postdose versus predose fingerprints, most changes at the annotated metabolite level were attributable to fasting metabolomics or potential confounders. ^1^H-NMR spectroscopy identified neither a short-term voriconazole-specific signature nor patterns or metabolites potentially reflecting acute CYP3A4 inhibition. Our study emphasizes crucial significance of strict standardization of fasting time and minimization of confounder influences by clinical trial design as well as selection of adequate baselines and high-resolution analytical techniques in pharmacometabolomics research, especially for biomarker discovery.

## 1. Introduction

Voriconazole is a second-generation, broad-spectrum triazole included on the WHO essential medicines list [1,2] and a cornerstone of prevention and treatment of invasive and potentially life-threatening fungal infections, especially in high-risk patient populations [3]. The treatment with voriconazole is associated with several challenges due to its complex pharmacokinetics and high interindividual variability [4] as well as relevant side effects [1]. Voriconazole is metabolized by cytochrome P450 (CYP) isozymes, particularly by CYP2C19 and CYP3A4, and is also a potent inhibitor of these enzymes, which predisposes it to clinically relevant drug–drug interactions. These drug–drug interactions can be associated with changed exposure of interacting drugs and voriconazole itself, therefore influencing therapeutic efficacy as well as the risk of side effects [4]. Being a major isozyme in human liver, CYP3A4 can metabolize both exogenous (e.g., drugs) and endogenous (e.g., cholesterol, steroid hormones, bile acids, arachidonic acid, vitamin D) substrates [5,6]. Modulation of CYP3A4 activity resulting in changed metabolism/systemic concentrations of its (endogenous) substrates may lead to adverse effects. Therefore, the availability of easily measurable specific biomarkers reliably reflecting the extent of, e.g., CYP3A4 activity would support rapid therapeutic decisions and increase treatment efficacy and safety. Such biomarkers to be used in clinical routine are currently missing. Overall, a deeper mechanistical understanding of voriconazole’s (adverse) effects is of high clinical relevance. Pharmacometabolomics is a powerful tool in clinical pharmacological research to investigate the mechanisms of drug effects as well as to identify potential biomarkers or biomarker sets for treatment efficacy, safety, and prediction of individual drug response [7,8]. The ability to characterize metabolomic signatures of drug exposure depends on the resolution of specific analytical metabolomic techniques, e.g., proton nuclear magnetic resonance (^1^H-NMR) spectroscopy, gas chromatography or liquid chromatography–mass spectrometry (GC-MS, LC-MS, respectively). Pharmacometabolomic fingerprints encompass individual metabolomic phenotypes (“metabotypes”) predetermined by intrinsic (genetic) and extrinsic (environmental) factors—disease- and drug-specific patterns, including also interaction patterns with drug-metabolizing enzymes such as, e.g., CYP450 isozymes, drug transporters and the microbiome, as well as alterations of the metabolic state (e.g., fasting). Therefore, interpretation of pharmacometabolomics data to distinguish drug-specific effects from confounder influences poses a major challenge for a vigorous study design. We explored a ^1^H-NMR spectroscopy-based untargeted urine pharmacometabolomics study in a pharmacokinetic clinical trial with 16 healthy participants, aiming to investigate: 1. the urinary metabolomic fingerprint of voriconazole 4 h after its administration in four single oral doses (50 mg, 100 mg, 200 mg, and 400 mg), 2. the metabolomic signature potentially reflecting acute CYP3A4 inhibition by voriconazole, which could be further investigated for characterization of biomarkers for therapeutic decision making in clinical practice, and 3. potential metabolomic signatures of confounders. In the main, pharmacokinetics evaluation of the trial, published earlier, significantly reduced midazolam and omeprazole clearances due to respective CYP3A4 and CYP2C19 inhibition were shown under all given voriconazole doses, with the highest decrease at 400 mg [9]. Therefore, we expected corresponding pharmacological effects on the metabolomics level. There are several human and animal metabolomics studies investigating voriconazole-induced hepatotoxicity in plasma, emphasizing the essential role of metabolomics technology in mechanistically clarifying drug-/voriconazole-induced effects [10,11]. To the best of our knowledge, this is the first clinical trial on untargeted urine pharmacometabolomics of voriconazole in humans.

## 2. Results

From around 150 metabolites, assessed by ^1^H-NMR spectroscopy (Table S1), the following 24 metabolites (16%) showed concentrations above zero in urine samples after creatinine normalization, independent of the treatment day: creatinine, methanol, alanine, betaine, creatine, glycine, N,N-dimethylglycine, N-isovaleroylglycine, proline betaine, valine, hippuric acid, acetic acid, citric acid, formic acid, succinic acid, tartaric acid, pantothenic acid, acetoacetic acid, acetone, oxaloacetic acid, pyruvic acid, caffeine, oxypurinol, and uracil. Only nine metabolites (representing 38% of the quantified metabolites pool or 6% of the entire metabolites pool) showed statistically significant changes (*p* values with false discovery rate (FDR) correction, presented in detail below), when values at predose were compared with the values 4 h postdose.

### 2.1. Predose and Postdose Metabolomic Fingerprints

Identified potential important confounders in our study were fasting duration (in the predose metabolomics—overnight fasting, and in the postdose metabolomics—extension of fasting duration, the individual overnight fasting plus 4 h), microdosed midazolam and omeprazole, sodium bicarbonate (in the preparation of omeprazole), body mass index (BMI), age, and sex.

To investigate the drug-specific patterns and to distinguish potential metabolomic signatures of confounders, we compared metabolomics fingerprints in four data settings:

- V1T0 versus V1T1 (first or baseline metabolomics day, before and after microdosed midazolam and omeprazole administration);
- V2T0 versus V2T1 (second metabolomics day, before and after administration of microdosed midazolam and omeprazole, and voriconazole);
- V1T1 versus V2T1 (postdose metabolomics on both treatment days);
- V1T0 versus V2T0 (baseline or predose metabolomics on both treatment days).

#### 2.1.1. Baseline Metabolomics V1T0 Versus V1T1

**Figure 1:**
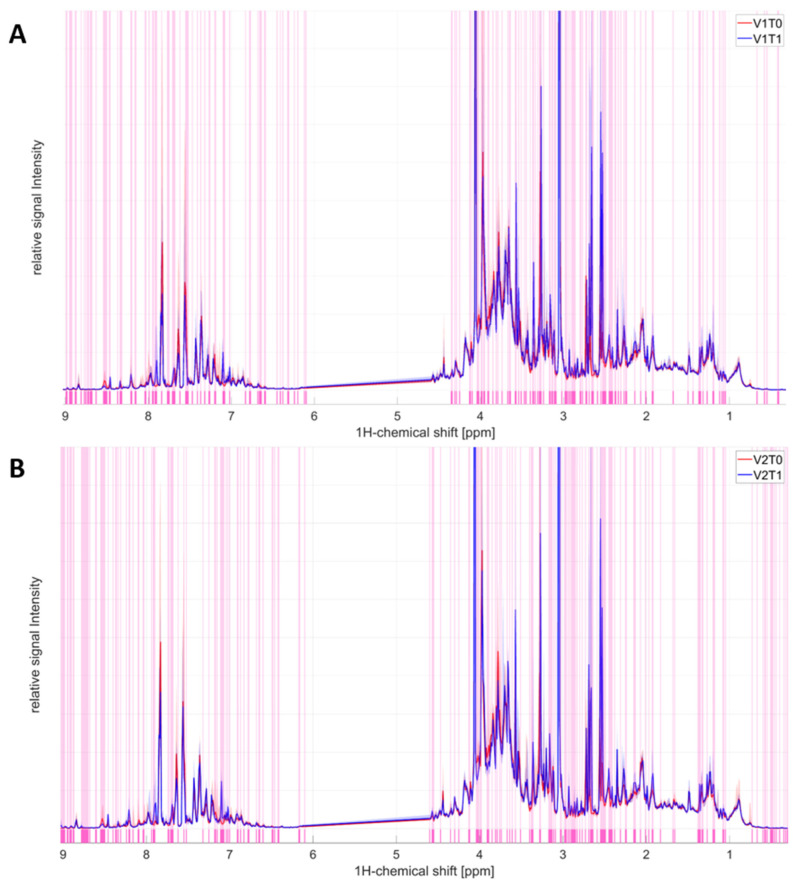
Univariate analysis of the full ^1^H-NMR urinary spectrum for both treatment days, predose and postdose settings. (**A**) Full ^1^H-NMR urinary spectrum (0.8–9.0 ppm) before (V1T0) and after (V1T1) the administration of the microdosed midazolam and omeprazole (corresponding to the overnight and postdose/plus 4 h fasting time). (**B**) Full ^1^H-NMR urinary spectrum (0.8–9.0 ppm) before (V2T0) and after (V2T1) administration of voriconazole and the microdosed midazolam and omeprazole (corresponding to the overnight and postdose/plus 4 h fasting time). The discriminating region between the groups is highlighted in light pink, revealed by the Kruskal–Wallis test (*p* < 0.05). The median of each group is represented by a line (T0 is red, T1 is blue), and the corresponding light color area represents the 5–95% percentile for each group. V1 = visit 1, V2 = visit 2, predose timepoint 0 = T0, postdose timepoint 1 = T1.

**Figure 2:**
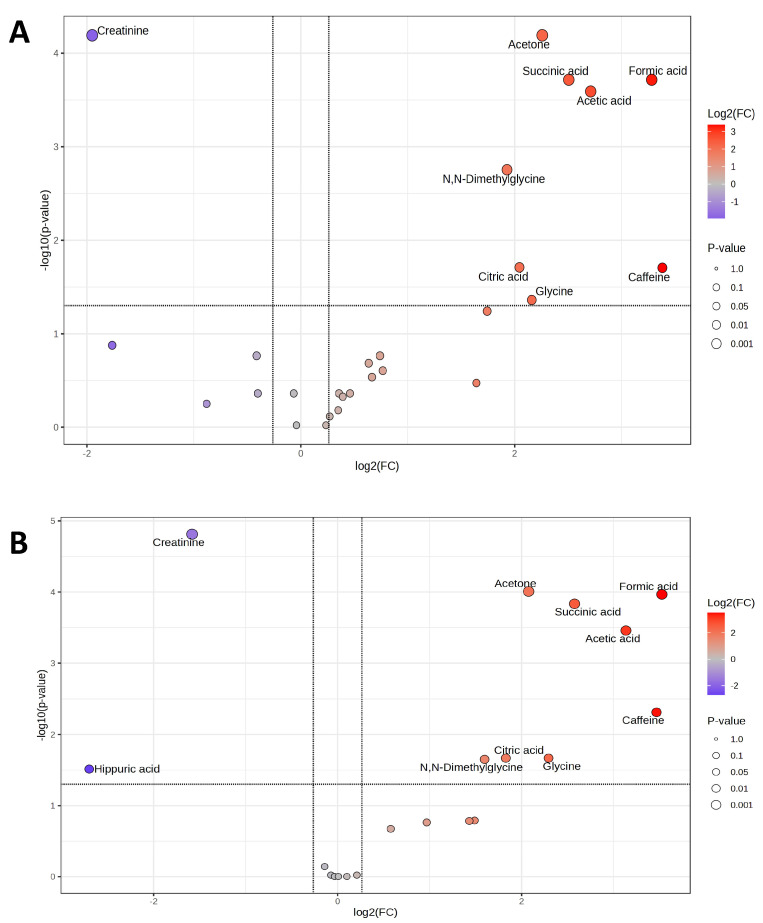
Volcano plots of two comparison settings, reflecting fold changes in urinary metabolite concentrations and the corresponding statistical significance. (**A**): Comparison of predose (timepoint 0, T0) and postdose (timepoint 1, T1) metabolomics patterns at visit 1 (V1). (**B**): Comparison of predose (T0) and postdose (T1) metabolomics patterns at visit 2 (V2). *X*-axis: log2(FC), *Y*-axis: −log10(*p*). Thresholds for significance: FC ≥ 1.2 (vertical line), *p* (FDR) < 0.05 (horizontal line). Red points show increased urinary metabolites; blue/violet points show decreased urinary metabolites. The point size corresponds to statistical significance. FC: Fold change, FDR: False discovery rate.

On V1, where only microdosed midazolam and omeprazole were administered and no voriconazole, the prolongation of the individual fasting period by 4 h resulted in statistically significant differences in predose/postdose fingerprints (Figure 1A), including both annotated and unannotated metabolomics. The following statistically significant changes and their magnitudes (presented as log2(FC)) in several known metabolites were detected, presented as volcano plots (Figure 2A): decreased urine creatinine excretion (FC 1.95), and increased excretion of caffeine (FC 3.38), formic acid (FC 3.28), acetic acid (FC 2.71), succinic acid (FC 2.51), acetone (FC 2.26), glycine (FC 2.16), citric acid (FC 2.04), and N,N-dimethylglycine (FC 1.93). Table 1 presents further details on concentrations and fold changes of individual metabolites (increased, decreased) together with the corresponding adjusted *p* values (FDR).

#### 2.1.2. Voriconazole Metabolomics, V2T0 Versus V2T1

At V2, where microdosed drugs and voriconazole were administered, the prolongation of the individual fasting period by 4 h resulted in statistically significant differences comprising both annotated and unannotated metabolomics (Figure 1B). The following statistically significant changes and their magnitudes (log2(FC)) in known metabolites were detected, presented as volcano plots (Figure 2B): decreased excretion of hippuric acid (FC 2.70) and creatinine (FC 1.58) and increased excretion of formic acid (FC 3.53), caffeine (FC 3.47), acetic acid (FC 3.13), succinic acid (FC 2.58), glycine (FC 2.29), acetone (FC 2.08), citric acid (FC 1.83), and N,N-dimethylglycine (FC 1.6). Table 2 presents further details on individual concentrations and fold changes of metabolites after voriconazole administration (pooled for all dose groups) with the corresponding adjusted *p* values (FDR).

Interestingly, caffeine excretion was detected in one out of sixteen participants after overnight fasting and in nine out of sixteen participants at postdose fasting on both treatment days, likely related to coffee consumption after the first 2 h of fluid abstinence. Oxypurinol excretion postdose was numerically increased, however, it did not reach statistical significance (raw *p* = 0.090).

#### 2.1.3. Metabolomics of V1T1 Versus V2T1 and V1T0 Versus V2T0

Postdose metabolomics without and with voriconazole, pooled for all doses (V1T1 and V2T1), revealed nearly identical metabolic patterns with nearly the same magnitude of changes in the metabolite concentrations, assessed by linear mixed effect models adjusted for total fasting time. The time between predose and postdose sampling was 4 h in all participants. Nevertheless, the inter-visit fasting times were slightly, but statistically significantly, different. The predose fasting times (T0) at V1 and V2 were 11.4 ± 1.5 h (minimum 9 h, maximum 15 h) and 10.6 ± 1.3 h (minimum 8 h, maximum 13 h), respectively (*p* = 0.029 in paired *t*-test). Analogously, the postdose fasting times at V1 and V2 were slightly, but statistically significantly, different with 15.4 ± 1.5 h (minimum 13 h, maximum 19 h) and 14.6 ± 1.3 h (minimum 12 h, maximum 17 h), respectively (*p* = 0.027 in paired *t*-test). Therefore, this difference in the inter-visit fasting time was taken into account for comparison of corresponding metabolite concentrations using a linear mixed-effects model test adjusted for total fasting time. Table 3 and Figure S1A present the postdose data and volcano plot analysis of corresponding fold changes for V1T1 versus V2T1 data settings, respectively. Table 3 and Figure S1B present the predose data and volcano plot of corresponding fold changes of metabolites for V1T0 versus V2T0 data settings, respectively.

**Figure 3:**
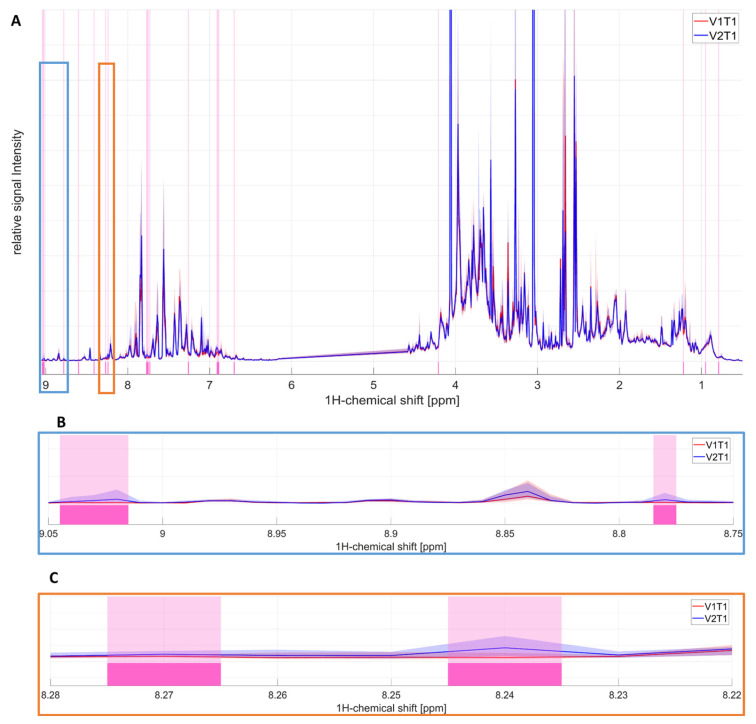
Univariate analysis of the full ^1^H-NMR urinary spectrum for the V1T1 and V2T1 groups. The discriminating region between the groups is highlighted in light pink, revealed by the Kruskal–Wallis test (*p* < 0.05). The median of each group is represented by a line (V1T1 is red, V2T1 is blue), and the corresponding light color area represents the 5–95% percentile for each group. (**A**) full ^1^H-NMR urinary spectrum (0.8–9.05 ppm). (**B**) A zoomed-in figure of a specific region illustrating the discrimination between the two groups (8.75–9.05 ppm, light blue border). (**C**) A zoomed-in figure of a specific region illustrating the discrimination between the two groups (8.22–8.28 ppm, orange border). The specific regions (**B**,**C**) are attributable to voriconazole.

The postdose fingerprints V1T1 and V2T1 were nearly identical (Figure 3A). A few specific annotated regions illustrating the discrimination between both fingerprints were identified to be attributable to voriconazole (Figure 3B,C).

No statistically significant differences were observed in the concentrations of the above-mentioned metabolites, when predose overnight metabolomics were compared between both treatment days (V1T0 and V2T0). The results are presented in Table 3, as well as in Figure S1B. The same was true for the corresponding fingerprints (Figure S2).

#### 2.1.4. Voriconazole Dose-Group Effects on Metabolite Concentrations

In the metabolomic signature of voriconazole exposure in four voriconazole dose groups, some dose-associated patterns of 50 mg to 400 mg dose groups were observed for valine and oxypurinol (Figure S3). When analyzing the excretion courses of both metabolites at other study timepoints, the observed concentration course seems to be related to individual excretion characteristics in participants assigned to the four voriconazole dose groups, rather than a voriconazole-related effect. Statistical analysis in the current case is not applicable due to small sample sizes per dose group and metabolite concentrations equaling zero in several participants. No dose-associated patterns were seen for other metabolites.

### 2.2. Multivariate Analysis

#### 2.2.1. Exploratory Analysis of Potential Confounders

The exploratory analysis with consideration of fasting duration at T1, age, sex, BMI, and voriconazole was conducted using LMM or MLR to estimate effect sizes for 4 h concentration differences (Supplementary Table S2). Residuals showed approximate normality across metabolites; minor tail deviations observed for isolated metabolites were not improved by log-transformation and were therefore analyzed on the original scale (Figure S4). Across metabolites, effect estimates and the corresponding *p* values indicated that sex and BMI could be confounders responsible for the observed changes in some metabolite concentrations in the predose–postdose analysis on both treatment days regardless of voriconazole (e.g., for N,N-dimethylglycine, acetic acid, and citric acid) (Supplementary Table S2). All other mentioned variables did not show any significant influence on the 4 h change in metabolite concentrations.

#### 2.2.2. Principal Component Analysis (PCA)

**Figure 4:**
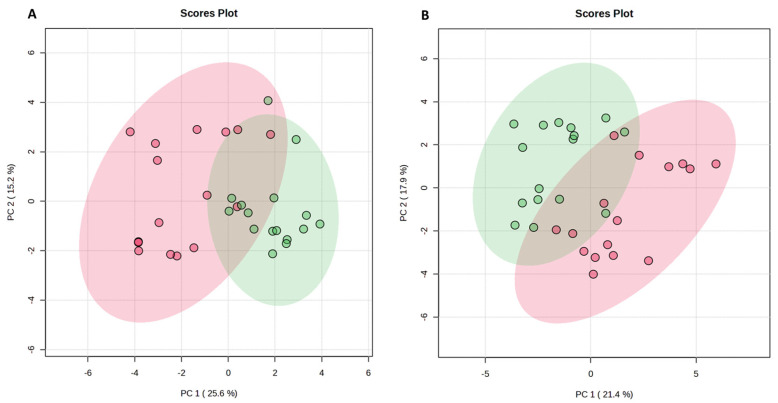
Principal component analysis (PCA) of urinary predose (T0) and postdose (T1) metabolomics on both treatment days, presented as scores plot. (**A**) Dataset on visit 2 (V2T0 versus V2T1). (**B**) Dataset on visit 1 (V1T0 versus V1T1). Green circles show predose and red circles postdose metabolomics. The ellipses in the corresponding colors outline the 95% confidence interval of each group. *X*- and *Y*-axes represent the first (PC 1) and the second (PC 2) principal components with respective variance percentages. T = timepoint, V = visit.

PCA revealed statistically significant differences and separation between predose and postdose metabolomics signatures on both treatment days, independent of voriconazole administration, and showed that the first (PC1) and second (PC2) principal components accounted for 40.8% of total variance in the dataset on visit 2 (Figure 4A) and for 39.3% of total variance in the dataset of visit 1 (Figure 4B). The subsequent PERMANOVA confirmed very strong, highly significant metabolic shifts comparing predose and postdose metabolomics signatures with F = 20.665, R^2^ = 0.408, *p* = 0.001 for visit 2 and with F = 16.084, R^2^ = 0.349, *p* = 0.001 for visit 1, based on 999 permutations in both analysis cases. PCA did not reveal any statistically significant differences between datasets of V1T1 versus V2T1 (F = 0.602; R^2^ = 0.020; *p* = 0.563) and V1T0 versus V2T0 (F = 1.334; R^2^ = 0.043; *p* = 0.279), based on 999 permutations in both analysis cases (Figure S5A,B).

## 3. Discussion

In our short-term untargeted urinary pharmacometabolomics evaluation of voriconazole, the predose and postdose fingerprints showed statistically significant differences across nearly all spectral regions on both treatment days, regardless of voriconazole administration. The comparison of both postdose fingerprints on V1 and V2 (without and with voriconazole, respectively) showed, however, no statistically significant metabolomic differences, except for a few regions at V2, which were attributable to voriconazole. In the annotated metabolomics dataset, the excretion of several metabolites was statistically significantly changed postdose versus predose on both treatment days to nearly the same extent, therefore, these differences were not specifically attributed to the short-term voriconazole exposure. Most of these metabolites are linked to (prolonged) fasting. Therefore, the fasting pattern, together with changes in some further metabolites discussed below, and their time-related longitudinal kinetics should be taken into account for the interpretation of pharmacometabolomics data in predose/postdose analysis settings. When expected systemic effects are minimal and group sizes are small, it is essential to keep variation (by any confounders) as low as possible. In our trial, we have strongly minimized potential preanalytical interference by employing a highly standardized urine sample collection and processing. The highly reproducible baseline predose metabolomics on both treatment days strengthen the findings of very low variability in excretion of individual metabolites in our study participants and, therefore, the validity of the corresponding findings even in our relatively small trial population. Especially in small investigational cohorts, the selection of adequate baselines (both, inter-day and intra-day) is of critical relevance for distinguishing drug effects from potential confounding influences. In the case of a solely intra-day pre-/postdose investigation design, potential confounders will be inadequately addressed, with the risk of assigning confounder-related metabolomic changes to drug-specific effects.

Under non-fasting conditions, glucose is the main energy source, while its excess is stored in the liver in the form of glycogen. During fasting, catabolic reactions in protein, lipid, and carbohydrate metabolism are triggered to keep blood glucose levels within a normal range [13,14]. After overnight fasting, liver glycogen is the main glucose source in plasma; however, its stores are rapidly exhausted within around 48 h. During prolonged fasting periods, the main glucose source is gluconeogenesis, i.e., glucose production from non-carbohydrate sources [15,16]. Overall, ketone bodies, lipids, and branched chain amino acids (BCAAs) are used from the body as alternative fuel sources in a fasting state [14].

### 3.1. Factors Potentially Influencing the Metabolomics Findings of the Study

**The selected metabolomics technology and biological matrix**. NMR technology, being a high-throughput technology with comparable high cost- and time-effectiveness and high reproducibility, seems to be a valuable pragmatic approach for untargeted metabolic characterization of urine [12]. Concentration determinations by NMR are usually very accurate due to direct measurement of the compound [12]. At the same time, there are some general limitations with NMR spectroscopy, particularly ^1^H-NMR spectroscopy, such as the comparably high detection threshold, impairing sensitivity for substances with low concentrations, as well as difficulties in precise identification of some metabolites, especially large molecules with complex chemical structures and spectral similarities between individual metabolites, e.g., different steroids [12,17,18]. Especially in the case of steroids (both endogenous and exogenous), which can be metabolized by the CYP3A4 isozyme, potentially reflecting the CYP3A4 activity via measuring corresponding metabolites in plasma and urine, MS- and MS/MS-based technology offers advantages over ^1^H-NMR spectroscopy [17]. Urine is an attractive biological matrix for metabolomics research, because it can be easily, non-invasively and repeatedly obtained in large amounts (therefore also representing valuable biofluid for (pharmaco)metabolomics research in pediatric populations), reflecting systemic metabolism. At the same time, the chemical complexity of urine makes it difficult for full and comprehensive understanding [12]. Considering the translatory potential and several above-mentioned advantages of both the metabolomics technology and the biofluid, we employed ^1^H-NMR spectroscopy for our untargeted urinary pharmacometabolomics study.

**Drug administration**. Being an indispensable life-saving drug, voriconazole is associated with a range of adverse effects [1]. In adult patients, voriconazole is administered as a loading dose of 400 mg BID in the first 24 h followed by a subsequent maintenance dose of 200 mg BID after the first 24 h. It is rapidly and nearly completely absorbed after oral administration, with maximum plasma concentrations (Cmax) achieved 1–2 h after dosing. It is metabolized in vivo predominantly by the hepatic isoenzymes CYP2C19 and CYP3A4, while less than 2% of the dose excreted unchanged in the urine. The terminal half-life of voriconazole depends on dose and is approximately 6 h with an oral 200 mg dose [1]. Administration of microdosed probe drugs for investigation of drug metabolism and pharmacokinetics is a well-recognized experimental approach [19]. Microdosed drugs are commonly not expected to cause relevant pharmacological effects that occur at their therapeutic doses. Omeprazole can inhibit the human organic cation transporter 2 (OCT2, SLC22A2OCT2) (responsible for uptake of organic compounds from the blood) and multidrug and toxin extrusion proteins such as MATE1 (SLC47A1) and MATE2-K (SLC47A2) (responsible for their secretion into the urine) in low micromolar concentration ranges [20,21,22]. However, omeprazole given in a microdose of 100 µg on both treatment days in our study and measured thereafter in plasma in the picomolar range [9] would not be expected to relevantly inhibit corresponding transporters and, therefore, to change the excretion of several substrate metabolites of these transporters to a relevant extent, as observed in our study.

In the main, pharmacokinetics evaluation of the trial, published earlier, significantly reduced midazolam and omeprazole clearances due to respective CYP3A4 and CYP2C19 inhibition were shown under all given voriconazole doses, with the highest decrease at 400 mg [9]. Therefore, we expected corresponding pharmacological effects on the metabolomics level. Around 30–50% of clinically used drugs are metabolized by the CYP3A family (particularly by CYP3A4) [23] and are, therefore, prone to clinically relevant drug–drug interactions with CYP3A4 inhibitors and inducers, associated with higher risk of adverse reactions or insufficient, subtherapeutic effects, respectively. Thus, reliable valid biomarkers for CYP3A4 activity, especially when easily measurable, acutely formed and having short half-lifes, would highly support therapeutic decisions, enabling corresponding rapid dose adjustments and enhancing treatment safety and efficacy. The urinary 6β-hydroxycortisol/cortisol ratio is suggested to be the best predictor of hepatic CYP3A activity under conditions of both maximal inhibition and maximal induction of the enzyme [24]. To measure this ratio, urine collection for at least 12 h is needed [24], representing limitations regarding easiness and suitability in a short-term setting in clinical practice. In our short-term study setting, no urinary metabolites (sets) were identified to be further validated as specific biomarker(s) for acute CYP3A4 inhibition, potentially guiding decision making in clinical practice for dose adjustments of drugs metabolized by CYP3A4. Nevertheless, we believe that every biological process in the body should leave its individual metabolic trace in body fluids, which could be measured by a suitable sensitive technique. Therefore, in future pharmacometabolomic studies, more sensitive techniques, such as LC-MS/MS, should be used for better detection of biochemical processes related to the enzymatic function profile of CYP3A4 and address this specific topic more successfully.

Participants had to ingest in total 200 mL sodium bicarbonate buffer (4.2%, *w*/*v*) to technically allow the administration of an omeprazole microdosing (100 mL was used in the omeprazole microdose preparation, and a further 100 mL 10 min prior to its administration) [9]. A 7-day sodium bicarbonate supplementation in patients with chronic kidney disease (CKD) was associated with statistically significant increased excretion of citric acid/isocitric acid (FC 1.50), 3-indoleacetic acid (FC 1.53), and glutaric acid (FC 1.30) in 24 h urine [25]. Therefore, sodium bicarbonate may have influenced, at least partly, the excretion of citric acid (and perhaps that of some other metabolites) in our trial.

### 3.2. Metabolomic Signatures, Changes in Individual Metabolites and Related Biomechanistic Explanations

To better understand the biomechanistic relationships of our findings and to explore the potential underlying cause–effect connections between our trial drug interventions (the pharmacometabolomics) and the impact of known potential confounders, we further discuss the formation/most likely sources, potential interfering factors, and biological interrelations as well as renal excretion mechanisms of several affected metabolites.

In our predose–postdose analysis setting, creatinine excretion was significantly decreased postdose in approximately the same magnitude of change on both treatment days regardless of voriconazole administration (Table 1 and Table 2). We assessed this effect most likely as a dilution effect after predose urine sampling, rather than having a biological background, because study drugs were administered with around 350 mL fluid and the participants were allowed to drink fluids after the initial fluid restriction for the first two hours after microdosed drug administration.

**Hippuric acid** excretion was statistically significantly decreased with prolongation of fasting time on the day with voriconazole administration and numerically (but not statistically significantly) decreased on the baseline treatment day (Table 1 and Table 2). Hippuric acid is a conjugation product of benzoic acid (originated from gut microflora or consumed with food) and glycine in the liver and kidney and is a common endogenous component of human urine [12]. It is linked to gut–liver metabolism as well as to diets rich in polyphenols such as fruits, vegetables, coffee, tea, and nuts [26]. Hippuric acid can also be produced from phenylalanine metabolism by gut microbiota [27]. Glycine availability is considered to be one of crucial rate-determining factors of hippurate production [26]. Hippuric acid is excreted in urine predominantly via tubular secretion, and tubulointerstitial impairment is associated with decreased hippurate excretion as a result of, e.g., reduced expression of organic anion transporters [26,28,29]. Remarkably, some intermediates of the tricarboxylic acid cycle (TCA) such as citrate and succinate may covary with the hippuric acid excretion in the same direction, linking hippuric acid formation and mitochondrial function in some cases [26]. However, in our trial setting, the excretion of these metabolites changed in different directions. Interestingly, hippuric acid excretion was relatively stable and not decreased in participants after overnight as well as 22 h and 24 h fasting [30]. Coffee consumption is known to increase hippuric acid excretion [31]. The decreased hippuric acid excretion observed in our study setting was, therefore, quite surprising, especially in participants who consumed coffee with the subsequent caffeine excretion in urine. This could be explained by insufficient time for production of hippuric acid between presumed coffee consumption and second/postdose spot urine sampling. We suggest that decreased excretion of hippuric acid in our trial may be linked to decreased availability of its sources to gut microflora (e.g., phenylalanine from dietary proteins, polyphenols) as well as decreased availability of glycine (increased glycine excretion in our trial). Voriconazole seems to have no influence on hippuric acid excretion, because we have not observed any relevant dose-related influences in the voriconazole dose groups.

**Citric acid, succinic acid, ketone bodies and BCAAs** are known fasting biomarkers, and their increased plasma concentrations as well as increased excretion in urine during fasting reflect adaptive mechanisms for maintenance of energy supply when glucose is unavailable [13,14,32,33,34]. Sodium bicarbonate buffer, administered in our study setting, may have, at least in part, increased citric acid excretion, discussed earlier. During fasting, increased production (and excretion) of acetic acid, being a short-chain fatty acid involved in carbohydrates, fats, and proteins, may be promoted by the breakdown of amino acids and fatty acid oxidation, generating acetyl-CoA and acetoacetate, which can be further metabolized into acetic acid and ketone bodies [32]. Remarkably, the urinary excretion of BCAAs (valine, leucine, isoleucine) was not statistically significantly changed with extension of fasting time in our setting. From the first impression, there was a decreasing tendency for valine excretion from 50 mg to 400 mg voriconazole dose groups (Figure S3A1). However, when analyzing valine excretion courses at other study timepoints, this seems to be related to individual excretion characteristics in participants assigned to the four voriconazole dose groups, rather than a voriconazole-related effect (Figure S3A2–A4). Overall, our data on **citric acid, succinic acid, acetic acid,** and **acetone** excretion are generally in good agreement with the literature data. We have not observed any short-term and dose-associated voriconazole effects, pointing out that voriconazole (in single-dose and short-term treatment settings) may not interfere with the TCA and ketone body metabolism.

Increased **glycine** and **N,N-dimethylglycine** excretions in our study may reflect adaptive metabolic changes during fasting as well, e.g., muscle protein catabolism to provide amino acids for energy or other metabolic needs (glycine) [35,36,37,38] and upregulated betaine turnover and (re)methylation reactions (N,N-dimethylglycine) [39,40,41,42,43,44]. The main mechanism for glycine urinary excretion (the so-called “glycine deportation system”), regulating systemic glycine exposure, is its conjugation with benzoic acid to form hippuric acid in the liver and (in lesser amounts) in the kidneys, which is then irreversibly filtered and excreted by the kidneys [45]. With this in mind, it was surprising that glycine and hippuric acid excretions in our trial were in opposite directions. Benzoic acid excretion was not changed with extension of fasting time in both treatment occasions independently of voriconazole in our trial (the creatinine-corrected values equaling zero). Moreover, we have not observed any relevant changes or tendencies in glycine or N,N-dimethylglycine excretion in different voriconazole dose groups.

For increased **formic acid** excretion postdose with prolonged fasting in our study, we can suggest both exogenous and endogenous mechanisms. Formic acid (or formate, the anion of formic acid) is a carboxylic acid which serves as a mediator of metabolic cross-talk between mammalian organism, the diet and the gut microbiome [36]. Glycine, choline, tryptophan, methionine, and serine catabolism, as well as cholesterol and sterol synthesis processes, are further potential sources of formate production in mammals [36]. Fifteen out of our sixteen participants had a measurable concentration of formate in urine in pre- and postdose samples. Formate can be formed from formaldehyde, which can be produced by demethylation of methylated molecules in drinks, foods, and drugs in the liver, e.g., from caffeine [36]. We cannot exclude caffeine as a potential additional exogenous source of formic acid in our study in at least some of our participants consuming coffee and excreting caffeine. However, we doubt that the short time after presumed coffee consumption would be sufficient for its complex production. Because glycine excretion was also increased in our trial, presumably increased glycine metabolism in a fasting state may be a potential endogenous source as well. We have not observed any voriconazole dose-related changes in formic acid excretion.

The observed statistically significantly increased **caffeine** excretion postdose with extension of fasting time on both treatment occasions is most likely not an endogenous biological effect but may be explained by coffee consumption in some of our participants (which occurred with varying frequency in the four dose groups) in the 2 h preceding urine sampling. Coffee consumption was not expected to affect the primary endpoint of our pharmacokinetics trial and, therefore, caffeine amount and timepoint of intake were not controlled. The time window for the absorption, distribution, metabolism, and renal excretion of caffeine was within 2 h of sampling. Caffeine (1,3,7-trimethylxanthine) is a plant alkaloid structurally similar to purines and has numerous physiological and pharmacological effects [46]. After its oral ingestion, it is completely absorbed with the Cmax being reached in around 30–60 min and mean plasma half-life in healthy individuals of 4–6 h (range 2–12 h) [47]. It is primarily metabolized in the liver by CYP1A2 to paraxanthine as its main metabolite (around 80% of caffeine), which is further metabolized to 5-acetylamino-6-formylamino-3-methyluracil and to 1-methylxanthine. Around one-third of 1-methylxanthine is excreted unchanged into urine, whereas the rest undergoes hydroxylation to 1-methyluric acid [48]. Further metabolites of caffeine are theobromine and theophylline, while around 2% of caffeine is excreted unchanged into urine [49]. Both glomerular filtration and renal tubular secretion (e.g., via OATs) are individually involved in their excretion [50]. 1-methylxanthine, but not caffeine and paraxanthine, can also inhibit OATs and, thus, may change the pharmacokinetics of several drugs, being excreted via OATs [50]. The creatinine-normalized concentration of theobromine, the only caffeine metabolite measured in our trial by ^1^H-NMR spectroscopy, equaled zero. Based on our own unpublished pharmacokinetics data on the time courses of individual caffeine metabolites’ elimination in urine as well as on available literature data [51], we believe that the time window of 0 to 2 h is most likely insufficient for the full formation and subsequent renal excretion of caffeine metabolites, especially in concentrations quantifiable by ^1^H-NMR spectroscopy. This is particularly conceivable for 1-methylxanthine, potentially capable of pharmacological inhibition of OATs with subsequent relevant changes in the excretion of susceptible metabolites in sufficiently high concentrations during regular caffeine consumption.

The origin of oxypurinol in urine in our trial is unclear. Oxypurinol is a xanthine analogue and is the active metabolite of the drug allopurinol with relatively long half-life of around 25 h in healthy people with normal renal function [52]. The conversion of allopurinol to oxypurinol is catalyzed most likely by the enzyme aldehyde oxidoreductase [53]. Both are known as xanthine oxidase inhibitors, preventing conversion of hypoxanthine to xanthine and xanthine to uric acid, therefore being used for treatment of gout [53]. No treatment with allopurinol was known or medically indicated in our healthy participants. Also, neither oxypurinol nor allopurinol was a known ingredient in any drug preparation used in our trial. Data on endogenous pathways for oxypurinol production is scarce. In a large urine metabolomics study in humans, reported oxypurinol concentrations were 13.3 (5.1–29.3) µmol/mol creatinine, while possible sources of oxypurinol (other than allopurinol) were not specifically mentioned [12]. Interestingly, oxypurinol was found in beer after the brewing process [54], suggesting diet as a possible source of oxypurinol in urine. In our trial, participants’ possible beer consumption on the day before our treatment visits was not documented. Literature data on other food products containing oxypurinol is scarce. Oxypurinol is excreted by the kidneys. The uric acid transporter URAT1 is reported to be involved in the reabsorption of oxypurinol from the urine, while an organic anion transporter (OAT4) seems to play a negligible role [55]. The oxypurinol urine concentrations in our trial at V2 were in the above-reported reference range in the dose groups 50–100–200 mg (5.0, 0.0 and 6.58 mmol/mol creatinine, respectively, measured in only one participant each in the 50 mg and 200 mg dose groups), however, the concentration was higher with 38.74 mmol/mol creatinine in the 400 mg group (measurable in all five participants of this group) (Figure S3B1). Due to small sample size, no meaningful statistical analysis was possible. When analyzing oxypurinol excretion courses at other study timepoints, the observed concentration course seems to be related to individual excretion peculiarities in participants assigned to the four voriconazole dose groups (Figure S3B2–B4), rather than a voriconazole-related effect. The CYP2C19 genotypes in participants of the 400 mg group did not show any specific influence on the oxypurinol excretion. Urinary uric acid was not measured in our trial setting. Therefore, the results in the 400 mg dose group should be interpreted with caution. There is no data from clinical practice that voriconazole may increase uric acid concentration in plasma or provoke an acute gout flare, which could serve as a sign of possible interaction of voriconazole with URAT1. The topic of oxypurinol urine excretion is intriguing and its possible sources (endogenous, exogenous/environmental and/or dietary) should be further addressed in appropriate future studies.

### 3.3. Limitations

We have to consider some limitations of our trial. First, this was a short-term clinical trial in healthy participants designed to investigate single-dose pharmacokinetic characteristics of voriconazole, especially effects on CYP3A4 and CYP2C19 activity, with corresponding short-term pharmacometabolomics evaluation. Therefore, metabolomics data from therapeutic voriconazole exposure over several days may yield different results. The investigation in healthy participants offers, at the same time, several advantages, because many confounders, such as comorbidity/disease-specific or comedication-specific metabolite patterns, could be excluded. The 4 h investigation time for voriconazole effects may be too short to comprise its systemic effects. However, the main (pharmacokinetics) study demonstrated clinically relevant CYP3A4 inhibition across all voriconazole doses in such a timeframe, suggesting that associated metabolomic changes should have been detectable with appropriate analytical tools. Second, while ^1^H-NMR spectroscopy may lack sensitivity to detect minor metabolic changes in our setting, its well-known advantages, including the translational potential, led us to select it for our current evaluation step. Future pharmacometabolomics studies should consider more sensitive platforms such as LC-MS/MS. Third, the small sample size with three to five participants in four voriconazole dose groups limits conclusions on dose-related effects. The multivariate analysis of potential confounder effects was limited due to the small sample size as well. In several models, singular fits or convergence issues occurred, limiting estimation of random effects and options to interpret the remaining models. In these cases, linear models were used instead, which may not fully account for within-subject correlation. Consequently, effect estimates and *p* values should be interpreted as descriptive measures. Considering the exploratory nature, feasibility and usual sample sizes in pharmacokinetics and -metabolomics research in the literature, the total sample size of 16 participants explores total metabolomic changes under voriconazole exposure (regardless of doses). The highly replicable baseline predose metabolomics on both treatment days with low variability in excretion of individual metabolites supports the interpretation of postdose results and mirrors the highly controlled setting of pharmacokinetic trials in general. Fourth, the availability of simultaneous plasma metabolomics data would allow us more comprehensive mechanistic interpretations of acute systemic effects of voriconazole and further support interpretation of urinary metabolomics results in our trial. Ideally, simultaneous urine and plasma metabolomics data should be pursued and used especially in pharmacometabolomics research, making the causative mechanistic interpretation of complex metabolic networks clearer and more meaningful and the chances of potential discoveries higher.

## 4. Materials and Methods

### 4.1. Clinical Trial

**Figure 5:**
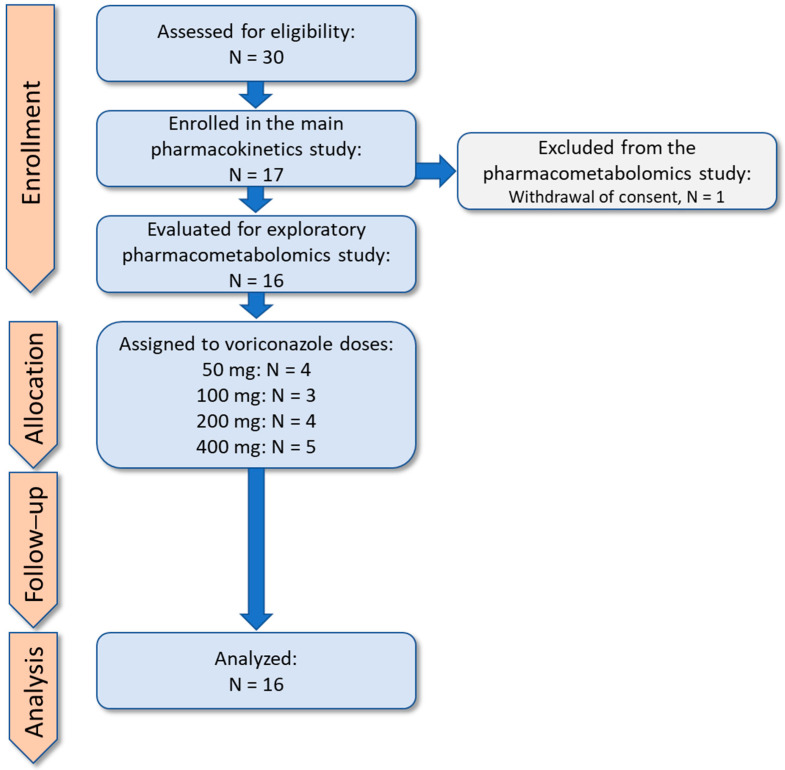
TREND flow diagram presenting the enrollment, allocation, follow–up and analysis in the clinical trial. mg = milligram, N = number, TREND = Transparent Reporting of Evaluations with Nonrandomized Designs.

This pharmacometabolomics study evaluated explorative objectives in the clinical trial on pharmacokinetics (clearance) of voriconazole via CYP3A and CYP2C19 using an in vivo approach with microdosed probe drugs for quantification of CYP3A4 (midazolam) and CYP2C19 (omeprazole) activity in 17 healthy adults, published earlier [9]. The clinical trial protocol was approved by the Competent Authority in Germany (BfArM, Bonn, EudraCT No: 2020-001017-20) and the responsible Ethics Committee of the Medical Faculty of Heidelberg University on 13 July 2020 (ethical approval code: AFmo-431/2020). One participant has withdrawn his consent for use of his biological samples for further investigations after publication of the main trial. In summary, 16 healthy participants (8 females and 8 males) with mean age of 30 years (minimum and maximum 22–50 years) and mean body mass index (BMI) of 24.2 kg/m^2^ (minimum and maximum 19.3–29.2 kg/m^2^) were evaluated for pharmacometabolomics (Figure 5). Regarding dietary habits, 15 participants had a balanced diet, one participant was vegan.

### 4.2. Study Design and Pharmacometabolomics Sampling

This single-center, fixed sequence, open-label, 4-arm, phase I trial in healthy participants included a screening visit, two treatment visits (V1, V2) 3–7 days apart, at which the samples for pharmacometabolomics were collected, and an end-of-trial visit [9] (please refer to the graphical abstract). On the first treatment day (baseline visit, V1), participants fasted overnight (water was allowed) and were administered oral microdosed midazolam (10 µg, in 100 mL tap water) and omeprazole (100 µg) to describe CYP3A and CYP2C19 clearance, respectively [9]. To prevent the degradation of uncoated omeprazole (OMEP^®^ 40 mg HEXAL powder for solution for infusion, Hexal AG, Holzkirchen, Germany) in gastric acid, the powder was dissolved in 100 mL of normal saline and 250 µL of the solution was further diluted in 100 mL of sodium bicarbonate buffer (4.2%, *w*/*v*). Additionally, participants drank 100 mL of sodium bicarbonate buffer 10 min prior to oral administration of the omeprazole microdose. Spot midstream urine samples were collected in 8.5 mL urine tubes (Urine Monovette^®^, Z, 8.5 mL, cap yellow, Sarstedt, Germany) around 1 h before (predose, timepoint T0) and around 4 h after microdosed drug administration (postdose, T1). Fluid intake was prohibited during the first 2 h after the trial medication administration and food for the first 4 h. After the first 2 h of fluid restriction (timeframe 2 h to 4 h postdose), participants were allowed to drink fluids, including coffee, however, the amount and the timepoint of the intake were not documented because they were not relevant to the primary and secondary endpoints of the trial. On the second treatment day (V2), participants received a single oral dose of either 50 mg, 100 mg, 200 mg, or 400 mg voriconazole (Voriconazol Hexal^®^ 50 mg film tablets, Hexal AG, Holzkirchen, Germany) followed by the administration of same probe drugs as on V1, timed one hour after voriconazole intake. Participants were sequentially assigned to voriconazole dose groups regardless of their CYP2C19 genotype, except for CYP2C19 poor metabolizers, who were always assigned 400 mg voriconazole (only one poor metabolizer was identified and included in this group). Four participants each were treated in the dose groups 50 mg and 200 mg, three participants in the 100 mg dose group and five participants in the 400 mg dose group. Spot midstream urine samples were collected in 8.5 mL urine tubes (Urine Monovette^®^, Z, 8.5 mL, cap yellow, Sarstedt, Germany) before (predose, timepoint T0) and 4 h after voriconazole administration (postdose, T1, corresponding to around 3 h after microdosed drug administration), before serving a meal, as at V1.

### 4.3. Sample Processing

In total, four urine samples per participant were collected. Sample collection, preanalytical processing, storage, and shipment followed established standard operating procedures, described elsewhere [56]. All urine samples were stored at room temperature for a maximum of 30 min before they were centrifuged at 2500× *g* and 4 °C for 5 min and stored at −80 °C until analysis. The samples were sent to the laboratory on dry ice under constant temperature monitoring.

### 4.4. Metabolomic Analysis, ^1^H-NMR Spectroscopy

Untargeted urinary metabolomics analysis of all samples was performed in full automation according to standard procedures on a Bruker IVDr System, as described previously [57]; a 600 MHz Bruker Avance III HD NMR spectrometer equipped with a SampleJet sample changer with sample cooling and a preheating station, a 5 mm inverse probe with z-gradient and automated tuning and matching, and a BCU-I in combination with Bruker’s body fluid NMR method package B.I.Methods 2.5 were used for fully automated acquisition and processing controlled by ICON NMR (Bruker BioSpin GmbH, Ettlingen, Germany).

Prior to measurement, urine samples were kept for 5 min inside the NMR probe head for temperature equilibration at 27 °C (300 K). Tuning and matching, locking, shimming, the optimization of the lock phase, and the calibration of the hard pulse at 90 °C were carried out automatically for the optimization of the NMR experimental conditions. Next, one-dimensional ^1^H-NMR nuclear Overhauser effect spectroscopy (NOESY, 32-scan) spectra were acquired by applying a standard pulse sequence with suppression of the water signal (Bruker pulse program library noesygppr1d, Bruker BioSpin GmbH, Ettlingen, Germany). Fourier transformation and fully automated phasing and baseline correction were carried out via the Bruker standard automation program APK0.NOE (Bruker BioSpin GmbH, Ettlingen, Germany). Spectra were quantitatively calibrated via the PULCON principle using an external reference sample (QuantRefC sample, Bruker BioSpin GmbH, Ettlingen, Germany) [58]. Trimethylsilyl propionic acid-d4 sodium salt (TSP) served as the reference peak. Two-dimensional 1H-NMR J-resolved spectroscopy (JRES, 2-scan) spectra were acquired.

Spectral Analysis: In total, up to 150 metabolites (Table S1), were quantified in ^1^H-NMR urine spectra using the Bruker IVDr analysis module B.I.QuantURe (Bruker BioSpin GmbH, Ettlingen, Germany). Raw NMR data and the absolute metabolite concentrations are available upon request.

### 4.5. Statistical Analysis

#### 4.5.1. Univariate Analysis

Descriptive statistics of affected metabolite concentrations in the investigated metabolomics settings were summarized using either the mean and standard deviation (SD) or median and 25–75% percentile depending on data distribution by normality testing using the D’Agostino and Pearson test and visualization with histograms. Metabolite concentrations were normalized to a relative concentration of creatinine and presented in mmol/mol to compensate for urine volume variations. Creatinine concentration is presented in mmol/L. Metabolites with over 50% missing values or values equaling zero after creatinine correction were excluded from the analysis.

Based on the trial design, participants served as their own controls in cases of comparisons of treatment effects without and with voriconazole (between-visit or interindividual comparison V1 versus V2) as well as in the predose and postdose analysis (intra-visit or within-subject comparison with timepoints T0 versus T1). The term “postdose” metabolic signatures in the text is related to the timepoint T1 during both treatment days (which is also the timepoint with extension of fasting time, comprising individual overnight fasting plus 4 h). The pooled metabolite concentration data over all voriconazole doses of 16 participants on V2 was compared with data without voriconazole administration (V1). Additionally, individual metabolite concentrations in each dose group were analyzed. With the small sample size per dose group (N = 5 in 400 mg, N = 4 in 50 mg and 200 mg, and N = 3 in 100 mg dose groups), and metabolite concentrations sometimes measured or changed in only one participant per group, as well as occasional concentrations equaling zero, no formal statistical analysis was performed on dose groups.

Fold change (FC) analysis, linear mixed-effect models (LMMs), and volcano plots were used to assess statistically significant differences and their magnitudes in the investigated groups. For FC analysis, metabolite concentrations were log10-transformed and Pareto-scaled prior to analysis [59]. The FC results are presented as log2 values. Raw *p* values < 0.05 were considered statistically significant. To reduce the number of potential false positive results due to a large number of metabolites and multiple comparisons, the Benjamini–Hochberg correction (false discovery rate, FDR) was applied to correct the raw *p* values, where appropriate. The metabolite fold change threshold of ≥1.2-fold (increases or decreases) was assessed as biologically significant, with adjusted *p* values (FDR) of ≤0.05 being statistically significant (paired groups, parametric tests). The threshold of ≥1.2-fold (increases or decreases) was also chosen to capture smaller (pharmacological) effects in metabolite concentrations, especially with regard to lower resolution of the ^1^H-NMR technique. Volcano plots depict the relationship between FC (log2) and statistical significance (−log_10_ *p* value) [59].

We employed the Kruskal–Wallis H test to identify significant differences in full metabolomic fingerprints among the different treatment groups. Each bin’s intensity was treated as an independent observation. The Kruskal–Wallis test compared the median intensities of these bins across groups to identify spectral bins with statistically significant differences between groups.

#### 4.5.2. Multivariate Analysis

##### Principal Component Analysis (PCA)

PCA with subsequent permutational analysis of variance (PERMANOVA) with 999 permutations was used as an unsupervised multivariate method for dimensionality reduction and data visualization to observe inter-group classification trends (clusters) (MetaboAnalyst 6.0 [59]) as well as to detect outliers.

##### Linear Mixed-Effect Models (LMMs)

For each metabolite, which showed statistically significant concentration changes in the predose–postdose FC analysis, we fitted a series of regression models with the 4 h metabolic change in concentration as the outcome and determined effect sizes for age, sex, voriconazole, the total fasting time at T1, and BMI as predictors. LMMs with a random effect were used when possible (based on the R package lme4) [60] to account for within-participant correlation (Equation (1)). *p* values were derived via Satterthwaite approximation. Model assumptions were evaluated using quantile–quantile (Q–Q) plots of residuals for all mixed-effects models.

(1) $$Y_{j}=\beta_{0}+\sum_{k=1}^{p}\beta_{k}X_{jk}+b_{0j}+\epsilon_{j}$$

Y_ij_ represents the 4 h change in metabolite concentration for participant j, β_0_ and β_k_ are the fixed-effect intercept and coefficients for the covariates, b_0j_ is a participant-specific random intercept accounting for within-subject correlation, and ϵ_ij_ is the residual error.

If a model was singular or failed to converge, likely due to the small sample size, a standard multivariable linear regression (MLR) model was applied (Equation (2)).

(2) $$Y_{j}=\beta_{0}+\sum_{k=1}^{p}\beta_{k}X_{jk}+\epsilon_{j}$$

Fixed-effect estimates, standard errors, and *p* values were obtained from the fitted models for each metabolite. Given the limited sample size and the exploratory scope of the multivariate analysis, no formal correction for multiple testing was applied in the LMM. The reported *p* values are descriptive with primary interpretation of the results focusing on the effect sizes and direction.

Statistical analyses were performed using Prism (version 10.2.3., GraphPad Software Inc., La Jolla, CA, USA), R (version 4.3.1, R Foundation for Statistical Computing, Vienna, Austria), and MetaboAnalyst 6.0 ([59], MetaboAnalyst-141 Pro-2025-1, Xia Lab, Montreal, QC, Canada).

No generative artificial intelligence (GenAI) has been used in any level of creation and visualization of this work.

## 5. Conclusions

^1^H-NMR spectroscopy identified neither a short-term voriconazole-specific signature nor patterns or metabolites reflecting acute CYP3A4 inhibition. The extension of fasting time by 4 h additionally to the overnight fasting, together with some other potential confounding influences, resulted in statistically significant changes in several metabolites in settings with and without voriconazole, indicating crucial significance of strict standardization of fasting time as well as selection of adequate baselines especially for pharmacometabolomics questions. Metabolomics techniques with higher resolution, e.g., LC-MS/MS, should be used for this specific question in future. Especially in pharmacometabolomics research and in small investigational populations, simultaneous assessments of plasma and urine with consideration of fasting metabolomics should be applied for better mechanistical understanding and distinguishing of drug-specific metabolomic effects from potential confounders, increasing, therefore, the chances of potential discoveries.

## Figures and Tables

**Table 1:** Changes in concentrations of urine metabolites in healthy participants at visit 1 (V1) before (T0) and 4 h after (T1) oral administration of microdosed midazolam and omeprazole.

Metabolite	HMDB ID	Chemical Taxonomy/Sub Class	Concentration, V1T0 (mM/M Creatinine)	Concentration, V1T1 (mM/M Creatinine)	Change	FC	log2 (FC)	*p* Value (FDR)	−log10 (*p*)	Literature Values [12], NMR mM/M Creatinine
Creatinine	HMDB00562	Amino acids, peptides, and analogues	14.50 (9.75–19.00) ^a^	3.50 (2.80–4.60) ^a^	↓	0.26	1.95	<0.001	4.19	14.74 ± 9.80
Caffeine	HMDB01847	Purines and purine derivatives	3.30 ± 13.20	44.06 ± 46.62	↑	10.42	3.38	0.020	1.71	1.20
Formic acid	HMDB00142	Carboxylic acids	3.42 ± 7.79	17.11 ± 8.91	↑	9.72	3.28	<0.001	3.72	26.80 (6.90–120.90)
Acetic acid	HMDB00042	Carboxylic acids	2.93 ± 4.24	15.53 ± 11.57	↑	6.55	2.71	<0.001	3.59	13.00 (2.50–106.00)
Succinic acid	HMDB00254	Dicarboxylic acids and derivatives	1.86 ± 2.94	9.28 ± 5.38	↑	5.68	2.51	<0.001	3.72	6.00 (0.30–33.30)
Acetone	HMDB01659	Carbonyl compounds	0.65 ± 1.17	5.36 ± 3.19	↑	4.78	2.26	<0.001	4.19	3.90 (0.80–17.60)
Glycine	HMDB00123	Amino acids, peptides, and analogues	52.10 (41.18–77.10)	121.7 (103.20–158.30)	↑	4.47	2.16	0.043	1.36	106.00 (44.00–300.00)
Citric acid	HMDB00094	Tricarboxylic acids and derivatives	120.20 (75.98–207.90)	433.6 (331.00–646.50)	↑	4.13	2.04	0.020	1.71	203.00 (49.00–600.00)
N,N-dimethylglycine	HMDB00092	Amino acids, peptides, and analogues	1.21 ± 2.77	5.59 ± 3.86	↑	3.81	1.93	0.002	2.75	4.40 (1.60–10.40)
Hippuric acid	HMDB00714	Benzoic acids and derivatives	203.70 ± 219.60	105.90 ± 146.70	n. s.	n. s.	n. s.	n. s.	n. s.	229.00 (19.00–622.00)

^a^ Absolute values of creatinine, mmol/L. FC = fold change, mM/M = millimole/mole, NMR = nuclear magnetic resonance spectroscopy, n. s. = not significant, T = timepoint, V = visit, the down arrow ↓ means decrease and the up arrow ↑ means increase in urinary excretion of the corresponding metabolites. Metabolites’ concentrations are presented as mean ± standard deviation (SD) or as median and (25–75% percentile), as appropriate.

**Table 2:** Changes in urine metabolites in healthy participants on visit day 2 before (T0) and 4 h after (T1) oral voriconazole administration in all dose groups (with background oral administration of microdosed midazolam and omeprazole, as at visit 1); no voriconazole-specific metabolic effects are detectable.

Metabolite	HMDB ID	Chemical Taxonomy	Concentration, V2T0 (mM/M Creatinine)	Concentration, V2T1 (mM/M Creatinine)	Change	FC	log2 (FC)	*p* Value (FDR)	−log10 (*p*)	Literature Values [12], NMR (mM/M Creatinine)
Hippuric acid	HMDB00714	Benzoic acids and derivatives	265.30 ± 163.80	176.00 ± 194.70	↓	0.15	2.70	0.030	1.51	229.00 (19.00–622.00)
Creatinine	HMDB00562	Amino acids, peptides, and analogues	11.00 (8.43–19.75) ^a^	4.20 (2.52–5.80) ^a^	↓	0.33	1.58	<0.001	4.81	14.74 ± 9.80
Formic acid	HMDB00142	Carboxylic acids	2.94 ± 5.30	18.41 ± 6.79	↑	11.52	3.53	<0.001	3.97	26.80 (6.90–120.90)
Caffeine	HMDB01847	Purines and purine derivatives	2.83 ± 11.30	45.29 ± 49.57	↑	11.07	3.47	0.005	2.31	1.20
Acetic acid	HMDB00042	Carboxylic acids	0.00 (0.00–4.80)	15.50 (8.73–21.03)	↑	8.78	3.13	<0.001	3.46	13.00 (2.50–106.00)
Succinic acid	HMDB00254	Dicarboxylic acids and derivatives	0.00 (0.00–5.80)	7.70 (6.33–11.48)	↑	5.96	2.58	<0.001	3.83	6.00 (0.30–33.30)
Glycine	HMDB00123	Amino acids, peptides, and analogues	62.06 ± 42.80	173.30 ± 121.90	↑	4.90	2.29	0.021	1.67	3.90 (0.80–17.60)
Acetone	HMDB01659	Carbonyl compounds	0.88 ± 1.37	5.05 ± 2.82	↑	4.23	2.08	<0.001	4.01	106.00 (44.00–300.00)
Citric acid	HMDB00094	Tricarboxylic acids and derivatives	162.70 ± 107.30	436.90 ± 214.00	↑	3.55	1.83	0.021	1.67	203.00 (49.00–600.00)
N,N-dimethylglycine	HMDB00092	Amino acids, peptides, and analogues	1.88 ± 2.89	5.53 ± 3.66	↑	3.03	1.60	0.022	1.65	4.40 (1.60–10.40)

^a^ Absolute values of creatinine, mmol/L. FC = fold change, mM/M = millimole/mole, NMR = nuclear magnetic resonance spectroscopy, T = timepoint, V = visit, the down arrow ↓ means decrease and the up arrow ↑ means increase in urinary excretion of the corresponding metabolites. Metabolites’ concentrations are presented as mean ± standard deviation (SD) or as median and (25–75% percentile), as appropriate.

**Table 3:** Changes in urine metabolites in healthy participants: A: on visit day 1 and visit day 2 with extended fasting time without (V1T1) and after voriconazole administration (V2T1); no voriconazole-specific metabolic effects are detectable; B: on visit day 1 before (V1T0) and visit day 2 before (V2T0) oral administration of any study drug (“baseline metabolomics”).

	A: V1T1 (After Microdosed Drugs) Versus V2T1 (After Microdosed Drugs and Voriconazole)			B: V1T0 Versus V2T0, “Baseline Metabolomics”		
Metabolite	Concentration, V1T1 (mM/M Creatinine)	Concentration, V2T1 (mM/M Creatinine)	Adjusted *p* Value *	Concentration, V1T0 (mM/M Creatinine)	Concentration, V2T0 (mM/M Creatinine)	Adjusted *p* Value *
Creatinine	3.50 (2.80–4.60)	4.20 (2.52–5.80)	0.593	14.50 (9.75–19.00)	11.00 (8.43–19.75)	0.207
Glycine	121.70 (103.20–158.30	122.8 (87.23–217.80)	0.144	52.10 (41.18–77.10)	67.35 (39.50–78.95)	0.790
N,N-dimethylglycine	6.35 (1.13–7.63)	6.30 (1.30–8.15)	0.915	0.00 (0.00–0.00)	0.00 (0.00–5.60)	0.527
Hippuric acid	105.90 ± 146.70	176.00 ± 194.70	0.231	203.70 ± 219.60	265.30 ± 163.80	0.182
Acetic acid	13.25 (6.73–21.05)	15.50 (8.73–21.03)	0.205	2.925 ± 4.243	2.03 ± 3.47	0.729
Citric acid	461.70 ± 181.50	436.90 ± 214.00	0.841	120.20 (75.98–207.90)	155.70 (81.83–211.70)	0.348
Formic acid	17.10 (14.23–21.25)	18.90 (14.50–23.63)	0.660	0.00 (0.00–0.00)	0.00 (0.00–7.73)	0.697
Succinic acid	8.55 (6.43–10.98)	7.70 (6.33–11.48)	0.410	1.86 ± 2.94	2.23 ± 3.02	0.425
Acetone	5.36 ± 3.19	5.05 ± 2.82	0.817	0.00 (0.00–0.00)	0.00 (0.00–00.00)	n. a.
Caffeine	44.06 ± 46.62	45.29 ± 49.57	0.806	0.00 (0.00–0.00)	0.00 (0.00–0.00)	n. a.

* Satterthwaite approximation, adjusted for total fasting time at T1 or T0. mM/M = millimole/mole, n. a. = not applicable, T = timepoint, V = visit. Metabolites’ concentrations are presented as mean ± standard deviation (SD) or as median and (25–75% percentile), as appropriate.

## Data Availability

The data supporting the findings of this study are presented within the article and its Supplementary Materials. The datasets used and/or analyzed during the current study are available from the corresponding author on reasonable request.

## Supplementary Materials

The following supporting information can be downloaded at: https://www.mdpi.com/article/10.3390/ijms27104468/s1.

## Abbreviations

The following abbreviations are used in this manuscript:

BCAA	branched chain amino acid
CYP3A4	cytochrome P450 (CYP) isozyme 3A4
CYP2C19	cytochrome P450 (CYP) isozyme 2C19
FC	fold change
FDR	false discovery rate
h	hour
^1^H-NMR	proton nuclear magnetic resonance spectroscopy
LMM	linear mixed-effect model
MATE	multidrug and toxin extrusion protein
min	minute
MLR	multivariable linear regression
log2	binary logarithm
µg	microgram
mg	milligram
OAT	organic anion transporter
OCT	organic cation transporter
TCA	tricarboxylic acid cycle
URAT	uric acid transporter
