# Supplementary material for: Interpretation of Pharmacometabolomics Results: Fingerprint of Drug Exposure or Confounder Effects? Insights from a Urinary Metabolomics Study with Voriconazole in Healthy Participants
Source: Int J Mol Sci. 2026 May 16;27(10):4468. doi: 10.3390/ijms27104468 (PMC13208054; doi:10.3390/ijms27104468)
Supplement: Supplementary file 1 [file ijms-27-04468-s001.zip › ijms-4283802 supplementary figure.pdf]

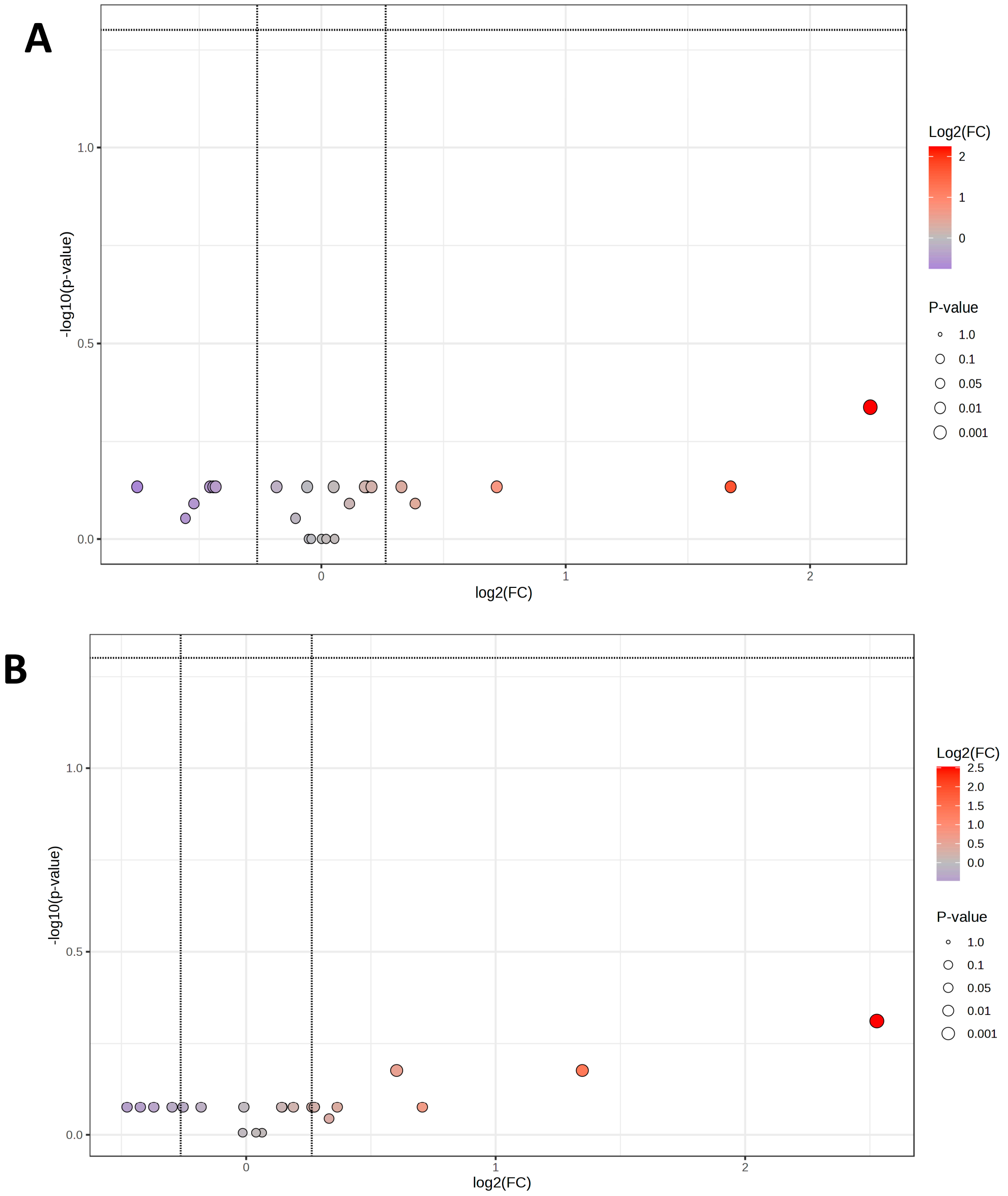

Figure S1. Volcano plots of two comparison settings, reflecting fold changes in urinary metabolite concentrations and the corresponding statistical significance. (A): Comparison of both postdose (T1) metabolomics patterns on visit 1 (V1) and visit 2 (V2). (B): Comparison of predose (T0) metabolomics patterns on V1 and V2. X-axis: log2(FC), Y-axis: -log10(p). Thresholds for significance: FC ≥ 1.2 (vertical line), P (FDR) < 0.05 (horizontal line). Red points show increased urinary metabolites; blue/violet points show decreased urinary metabolites. The point size corresponds to statistical significance. FC: fold change, FDR: false discovery rate, T = timepoint, V = visit.

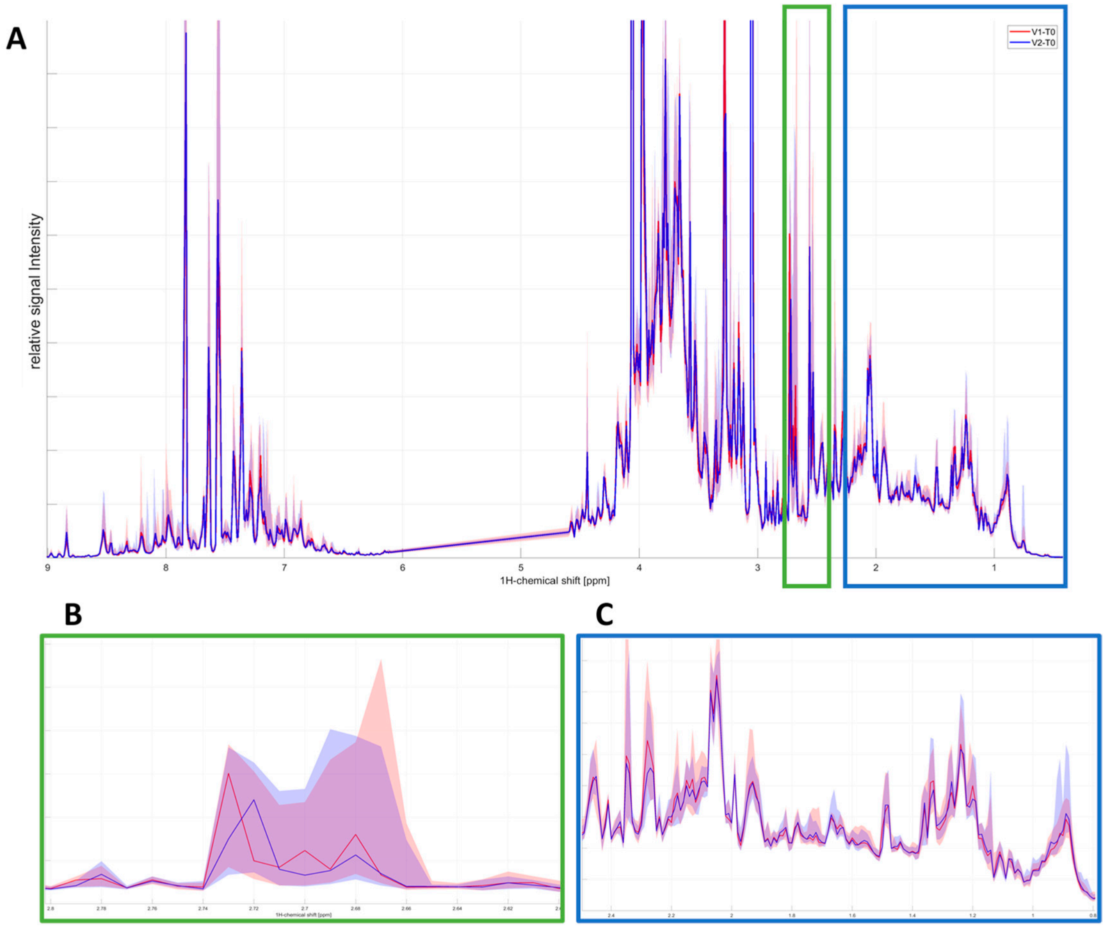

Figure S2. Univariate analysis of the full ^{1}H-NMR urinary spectrum for the V1T0 and V2T0 groups. The discriminating region between the groups is highlighted in light pink, revealed by the Kruskal-Wallis test ($P < 0.05$). The median of each group is represented by a line (V1T0 is red, V2T0 is blue), and the corresponding light color area represents the 5-95% percentile for each group. (**A**) Full ^{1}H-NMR urinary spectrum (0.8-9.0 ppm). (**B**) A zoomed-in figure of a specific region illustrating the discrimination between the two groups (2.6-2.8 ppm, green border). (**C**) A zoomed-in figure of the aliphatic region (0.8-2.5 ppm, light blue border). T = timepoint, V = visit.

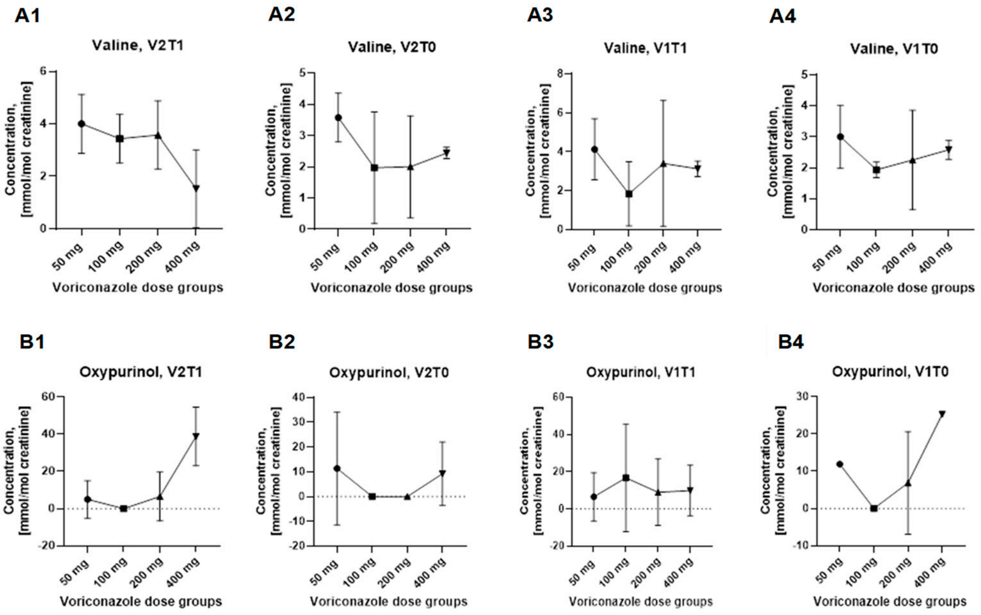

Figure S3. Concentration courses of valine (A1–A4) and oxypurinol (B1–B4) in four voriconazole dose groups. Figures A1 and A2 presents the postdose (T1) and predose (T0) courses of valine concentrations on visit 2 (after and before administration of voriconazole). Figures A3 and A4 present the individual courses of valine concentrations on visit 1 in participants, according to their later assignment to corresponding voriconazole dose groups (no voriconazole was administered on visit 1). Figures B1 and B2 present the postdose (T1) and predose (T0) courses of oxypurinol concentrations on visit 2 (after and before administration of voriconazole). Figures B3 and B4 present the individual courses of oxypurinol concentrations on visit 1 in participants, according to their later assignment to corresponding voriconazole dose groups (no voriconazole was administered on visit 1). T = timepoint, V = visit.

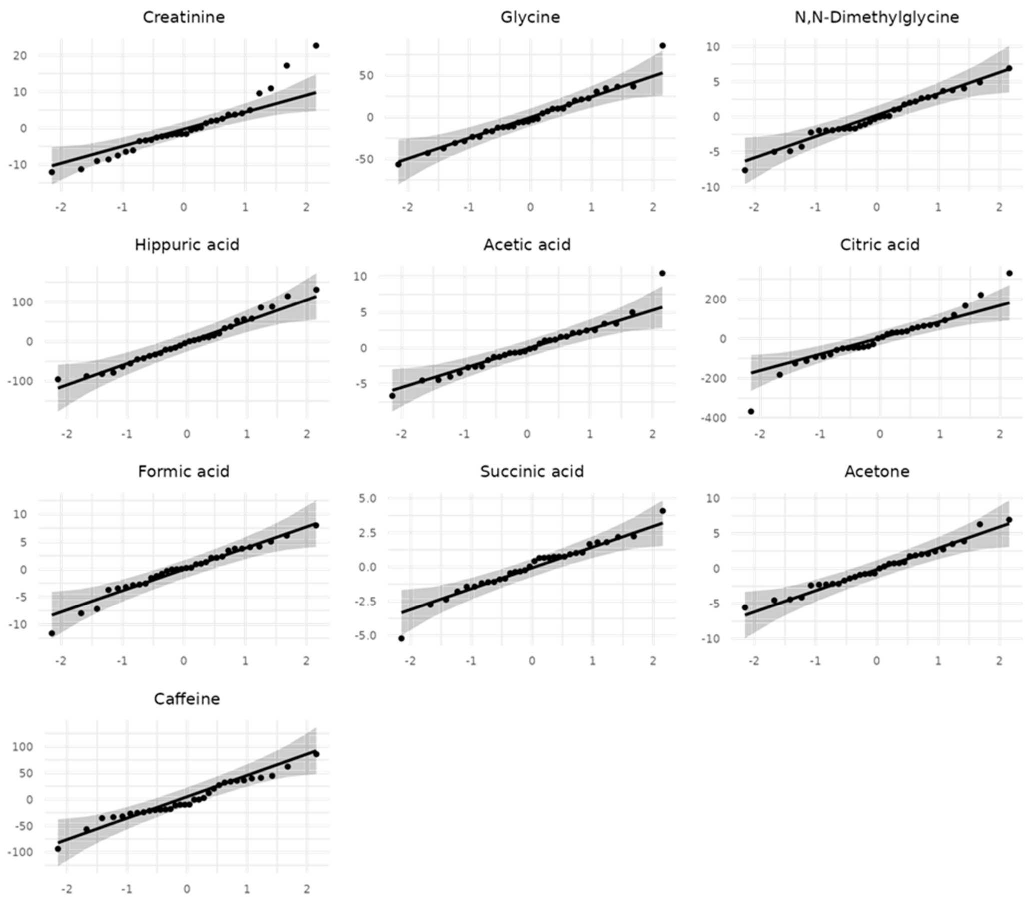

Figure S4. Quantile–quantile (Q-Q) plots of residuals form the fully adjusted mixed-effects model (Δ Metabolic conc. ~ age + sex + voriconazole dose + fasting time at T1 + BMI + (1|Participant ID)) for each metabolite. Shaded bands indicate 95% confidence intervals. Overall alignment with the reference line indicates approximate normality of residuals across metabolites.

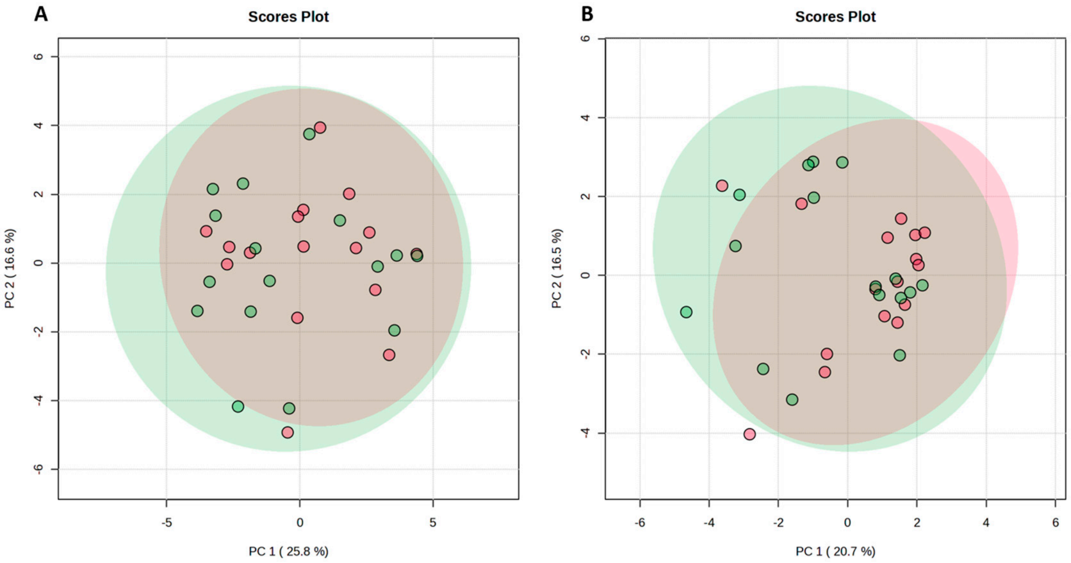

Figure S5. Principal component analysis of urinary between-day postdose and predose metabolomic profiles on both treatment days, presented as scores plot. (A): Postdose metabolomics on visit 1 and visit 2 (V1T1 versus V2T1 comparison). (B): Predose metabolomics on visit 1 and visit 2 (V1T0 versus V2T0 comparison). Green circles show V1 and red circles V2 metabolomics. The ellipses in the corresponding colors outline the 95% confidence interval of each group. X- and Y-axes represent the first (PC 1) and the second (PC 2) principal components with respective variance percentages. T = timepoint, V = visit.
