# Supplementary material for: Interpretation of Pharmacometabolomics Results: Fingerprint of Drug Exposure or Confounder Effects? Insights from a Urinary Metabolomics Study with Voriconazole in Healthy Participants
Source: Int J Mol Sci. 2026 May 16;27(10):4468. doi: 10.3390/ijms27104468 (PMC13208054; doi:10.3390/ijms27104468)
Supplement: Supplementary file 1 [file ijms-27-04468-s001.zip › Table S2_Structured effect sizes of potential confounders on metabolites.pdf]

Table S2. Structured effect sizes of potential confounders per metabolite. Model formulas with (lmer) and (lm) correspondent to linear mixed-effects or multivariate linear regressions models. Effect sizes for 4 h concentration difference of the following metabolites:

Creatinine

Equation	Age	Sex (male)	Voriconazole dose	Fasting time at T1	BMI
Δ Conc. ~ age + sex + Wmg_VRC + fastingtime_T1 + bmi (lmer)	-0.135 (±0.168), p-value: 0.43749	3.237 (±3.171), p-value: 0.32652	-0.013 (±0.011), p-value: 0.24355	0.236 (±1.183), p-value: 0.84359	0.245 (±0.555), p-value: 0.66625
Δ Conc. ~ age + sex + Wmg_VRC + fastingtime_T1 (lm)	-0.132 (±0.164), p-value: 0.42747	3.517 (±3.041), p-value: 0.25756	-0.012 (±0.011), p-value: 0.26238	0.382 (±1.117), p-value: 0.73517	
Δ Conc. ~ age + sex + Wmg_VRC + bmi (lm)	-0.129 (±0.161), p-value: 0.43016	3.024 (±2.918), p-value: 0.30919	-0.014 (±0.010), p-value: 0.19155		0.276 (±0.521), p-value: 0.60085
Δ Conc. ~ age + sex + Wmg_VRC (lm)	-0.122 (±0.158), p-value: 0.44926	3.210 (±2.859), p-value: 0.27105	-0.013 (±0.010), p-value: 0.20818		
Δ Conc. ~ age + sex + fastingtime_T1 + bmi (lmer)	-0.111 (±0.170), p-value: 0.52655	4.171 (±3.135), p-value: 0.20763		0.655 (±1.145), p-value: 0.57313	0.096 (±0.552), p-value: 0.86507
Δ Conc. ~ age + sex + fastingtime_T1 (lm)	-0.110 (±0.164), p-value: 0.50563	4.253 (±2.988), p-value: 0.16573		0.697 (±1.089), p-value: 0.52751	
Δ Conc. ~ Wmg_VRC (lm)			-0.013 (±0.010), p-value: 0.18223		
Δ Conc. ~ fastingtime_T1 (lmer)				0.177 (±1.036), p-value: 0.86538	
Δ Conc. ~ bmi (lmer)					0.211 (±0.521), p-value: 0.69164

bmi = body mass index (kg/m^2); Δ Conc. = concentration difference; VRC = voriconazole; T1 = timepoint 1; Wmg = voriconazole dose.

Glycine

Equation	Age	Sex (male)	Voriconazole dose	Fasting time at T1	BMI
Δ Conc. ~ age + sex + Wmg_VRC + fastingtime_T1 + bmi (lmer)	3.249 (±1.996), p-value: 0.13046	-62.973 (±37.475), p-value: 0.1169	0.050 (±0.061), p-value: 0.42045	-13.196 (±9.736), p-value: 0.19079	-6.263 (±6.522), p-value: 0.35618
Δ Conc. ~ age + sex + Wmg_VRC + fastingtime_T1 (lmer)	3.161 (±1.999), p-value: 0.13815	-68.085 (±37.198), p-value: 0.08861	0.043 (±0.060), p-value: 0.48598	-14.775 (±9.572), p-value: 0.13803	
Δ Conc. ~ age + sex + Wmg_VRC + bmi (lmer)	2.947 (±1.866), p-value: 0.14011	-49.089 (±33.860), p-value: 0.17272	0.083 (±0.058), p-value: 0.17502		-7.662 (±6.052), p-value: 0.22952
Δ Conc. ~ age + sex + Wmg_VRC (lmer)	2.791 (±1.901), p-value: 0.16597	-53.439 (±34.397), p-value: 0.14446	0.078 (±0.058), p-value: 0.20026		
Δ Conc. ~ age + sex + fastingtime_T1 + bmi (lmer)	3.218 (±2.003), p-value: 0.13525	-68.799 (±36.971), p-value: 0.08716		-16.907 (±8.666), p-value: 0.06557	-5.541 (±6.491), p-value: 0.4109
Δ Conc. ~ age + sex + fastingtime_T1 (lmer)	3.141 (±1.989), p-value: 0.13889	-72.543 (±36.504), p-value: 0.06786		-17.747 (±8.570), p-value: 0.05139	

Equation	Age	Sex (male)	Voriconazole dose	Fasting time at T1	BMI
Δ Conc. ~ Wmg_VRC (lmer)			0.080 (±0.058), p-value: 0.18587		
Δ Conc. ~ fastingtime_T1 (lmer)				-14.031 (±8.626), p-value: 0.11927	
Δ Conc. ~ bmi (lmer)					-7.376 (±6.372), p-value: 0.2664

N,N-Dimethylglycine

Equation	Age	Sex (male)	Voriconazole dose	Fasting time at T1	BMI
Δ Conc. ~ age + sex + Wmg_VRC + fastingtime_T1 + bmi (lm)	0.033 (±0.070), p-value: 0.64371	-0.230 (±1.399), p-value: 0.87091	0.007 (±0.005), p-value: 0.16003	0.959 (±0.604), p-value: 0.12437	-0.816 (±0.231), **p-value: 0.00157 ***
Δ Conc. ~ age + sex + Wmg_VRC + fastingtime_T1 (lmer)	0.025 (±0.089), p-value: 0.77856	-1.322 (±1.719), p-value: 0.45422	0.003 (±0.005), p-value: 0.5464	0.399 (±0.701), p-value: 0.57393	
Δ Conc. ~ age + sex + Wmg_VRC + bmi (lm)	0.054 (±0.070), p-value: 0.45255	-1.254 (±1.276), p-value: 0.33473	0.004 (±0.005), p-value: 0.36763		-0.712 (±0.228), **p-value: 0.00423 ***
Δ Conc. ~ age + sex + Wmg_VRC (lmer)	0.035 (±0.085), p-value: 0.68486	-1.722 (±1.531), p-value: 0.28056	0.002 (±0.005), p-value: 0.66496		
Δ Conc. ~ age + sex + fastingtime_T1 + bmi (lm)	0.023 (±0.071), p-value: 0.75031	-0.841 (±1.361), p-value: 0.54168		0.647 (±0.575), p-value: 0.27051	-0.735 (±0.229), **p-value: 0.0034 ***
Δ Conc. ~ age + sex + fastingtime_T1 (lmer)	0.021 (±0.087), p-value: 0.8091	-1.587 (±1.631), p-value: 0.34671		0.256 (±0.653), p-value: 0.69855	
Δ Conc. ~ Wmg_VRC (lmer)			0.003 (±0.005), p-value: 0.59421		
Δ Conc. ~ fastingtime_T1 (lmer)				0.504 (±0.583), p-value: 0.39459	
Δ Conc. ~ bmi (lm)					-0.696 (±0.222), **p-value: 0.0038 ***

* Statistically significant P values are in bold.

Hippuric acid

Equation	Age	Sex (male)	Voriconazole dose	Fasting time at T1	BMI
Δ Conc. ~ age + sex + Wmg_VRC + fastingtime_T1 + bmi (lmer)	0.202 (±2.651), p-value: 0.94049	-12.995 (±50.968), p-value: 0.80262	-0.100 (±0.113), p-value: 0.39091	-5.182 (±17.076), p-value: 0.76412	9.842 (±8.706), p-value: 0.28059
Δ Conc. ~ age + sex + Wmg_VRC + fastingtime_T1 (lmer)	0.308 (±2.645), p-value: 0.90933	-2.728 (±50.163), p-value: 0.95742	-0.077 (±0.113), p-value: 0.50487	-0.727 (±16.798), p-value: 0.96583	
Δ Conc. ~ age + sex + Wmg_VRC + bmi (lmer)	0.086 (±2.548), p-value: 0.97365	-7.507 (±46.226), p-value: 0.87366	-0.086 (±0.102), p-value: 0.41077		9.287 (±8.260), p-value: 0.28259

Equation	Age	Sex (male)	Voriconazole dose	Fasting time at T1	BMI
Δ Conc. ~ age + sex + Wmg_VRC (lmer)	0.290 (±2.570), p-value: 0.91188	-2.000 (±46.481), p-value: 0.96633	-0.075 (±0.102), p-value: 0.47235		
Δ Conc. ~ age + sex + fastingtime_T1 + bmi (lmer)	0.289 (±2.617), p-value: 0.91387	-2.275 (±48.991), p-value: 0.96368		1.286 (±15.526), p-value: 0.93464	8.488 (±8.470), p-value: 0.33669
Δ Conc. ~ age + sex + fastingtime_T1 (lmer)	0.360 (±2.595), p-value: 0.8918	4.801 (±48.162), p-value: 0.92207		4.079 (±15.365), p-value: 0.79278	
Δ Conc. ~ Wmg_VRC (lmer)			-0.076 (±0.100), p-value: 0.45454		
Δ Conc. ~ fastingtime_T1 (lmer)				5.461 (±14.104), p-value: 0.7014	
Δ Conc. ~ bmi (lmer)					8.605 (±7.639), p-value: 0.27893

Acetic acid

Equation	Age	Sex (male)	Voriconazole dose	Fasting time at T1	BMI
Δ Conc. ~ age + sex + Wmg_VRC + fastingtime_T1 + bmi (lmer)	-0.336 (±0.289), p-value: 0.26697	-12.350 (±5.373), **p-value: 0.03847 ***	0.012 (±0.007), p-value: 0.12467	0.685 (±1.185), p-value: 0.56998	-0.046 (±0.942), p-value: 0.96198
Δ Conc. ~ age + sex + Wmg_VRC + fastingtime_T1 (lmer)	-0.337 (±0.277), p-value: 0.246	-12.392 (±5.132), **p-value: 0.02982 ***	0.012 (±0.007), p-value: 0.12486	0.672 (±1.166), p-value: 0.57113	
Δ Conc. ~ age + sex + Wmg_VRC + bmi (lmer)	-0.321 (±0.286), p-value: 0.28369	-13.078 (±5.191), **p-value: 0.02688 ***	0.010 (±0.006), p-value: 0.13979		0.028 (±0.928), p-value: 0.97638
Δ Conc. ~ age + sex + Wmg_VRC (lmer)	-0.320 (±0.274), p-value: 0.26339	-13.066 (±4.963), **p-value: 0.02066 ***	0.010 (±0.006), p-value: 0.14113		
Δ Conc. ~ age + sex + fastingtime_T1 + bmi (lmer)	-0.344 (±0.284), p-value: 0.24842	-13.685 (±5.226), **p-value: 0.02141 ***		-0.154 (±1.121), p-value: 0.89198	0.120 (±0.920), p-value: 0.89805
Δ Conc. ~ age + sex + fastingtime_T1 (lmer)	-0.342 (±0.273), p-value: 0.23088	-13.599 (±4.998), **p-value: 0.0167 ***		-0.130 (±1.108), p-value: 0.9077	
Δ Conc. ~ Wmg_VRC (lmer)			0.011 (±0.006), p-value: 0.09945		
Δ Conc. ~ fastingtime_T1 (lmer)				0.130 (±1.117), p-value: 0.90873	
Δ Conc. ~ bmi (lmer)					-0.201 (±1.101), p-value: 0.85755

Citric acid

Equation	Age	Sex (male)	Voriconazole dose	Fasting time at T1	BMI
Δ Conc. ~ age + sex + Wmg_VRC + fastingtime_T1 + bmi (lm)	0.913 (±2.724), p-value: 0.74013	-148.798 (±54.593), **p-value: 0.01133 ***	-0.248 (±0.184), p-value: 0.18983	-17.933 (±23.560), p-value: 0.45341	-0.108 (±9.022), p-value: 0.99055
Δ Conc. ~ age + sex + Wmg_VRC + fastingtime_T1 (lm)	0.912 (±2.672), p-value: 0.73543	-148.950 (±52.095), **p-value: 0.00809 ***	-0.249 (±0.175), p-value: 0.16775	-18.013 (±22.169), p-value: 0.4236	
Δ Conc. ~ age + sex + Wmg_VRC + bmi (lm)	0.520 (±2.654), p-value: 0.84613	-129.649 (±48.072), **p-value: 0.01191 ***	-0.198 (±0.171), p-value: 0.2564		-2.057 (±8.584), p-value: 0.81242
Δ Conc. ~ age + sex + Wmg_VRC (lm)	0.464 (±2.599), p-value: 0.8595	-131.037 (±46.912), **p-value: 0.00931 ***	-0.205 (±0.166), p-value: 0.2275		
Δ Conc. ~ age + sex + fastingtime_T1 + bmi (lm)	1.272 (±2.752), p-value: 0.6475	-126.595 (±52.820), **p-value: 0.02373 ***		-6.616 (±22.338), p-value: 0.76936	-3.066 (±8.881), p-value: 0.73263
Δ Conc. ~ age + sex + fastingtime_T1 (lm)	1.268 (±2.708), p-value: 0.64333	-129.746 (±51.201), **p-value: 0.01715 ***		-8.292 (±21.458), p-value: 0.70211	
Δ Conc. ~ Wmg_VRC (lmer)			-0.141 (±0.176), p-value: 0.42964		
Δ Conc. ~ fastingtime_T1 (lm)				12.764 (±20.800), p-value: 0.54407	
Δ Conc. ~ bmi (lmer)					-5.528 (±9.243), p-value: 0.55936

Formic acid

Equation	Age	Sex (male)	Voriconazole dose	Fasting time at T1	BMI
Δ Conc. ~ age + sex + Wmg_VRC + fastingtime_T1 + bmi (lmer)	-0.089 (±0.218), p-value: 0.69206	-5.895 (±4.162), p-value: 0.17866	0.005 (±0.008), p-value: 0.52805	0.669 (±1.299), p-value: 0.61171	-0.694 (±0.715), p-value: 0.35016
Δ Conc. ~ age + sex + Wmg_VRC + fastingtime_T1 (lmer)	-0.097 (±0.216), p-value: 0.66106	-6.565 (±4.075), p-value: 0.129	0.004 (±0.008), p-value: 0.63754	0.405 (±1.276), p-value: 0.75337	
Δ Conc. ~ age + sex + Wmg_VRC + bmi (lmer)	-0.074 (±0.212), p-value: 0.73313	-6.610 (±3.842), p-value: 0.1109	0.004 (±0.008), p-value: 0.64574		-0.622 (±0.687), p-value: 0.38304
Δ Conc. ~ age + sex + Wmg_VRC (lmer)	-0.087 (±0.209), p-value: 0.68373	-6.974 (±3.788), p-value: 0.08866	0.003 (±0.008), p-value: 0.70687		
Δ Conc. ~ age + sex + fastingtime_T1 + bmi (lmer)	-0.093 (±0.213), p-value: 0.67015	-6.484 (±3.978), p-value: 0.12644		0.312 (±1.170), p-value: 0.79241	-0.620 (±0.691), p-value: 0.38663
Δ Conc. ~ age + sex + fastingtime_T1 (lmer)	-0.100 (±0.211), p-value: 0.64482	-6.960 (±3.911), p-value: 0.0965		0.153 (±1.156), p-value: 0.89562	
Δ Conc. ~ Wmg_VRC (lmer)			0.005 (±0.007), p-value: 0.55327		
Δ Conc. ~ fastingtime_T1 (lmer)				0.581 (±1.117), p-value: 0.60742	

Equation	Age	Sex (male)	Voriconazole dose	Fasting time at T1	BMI
Δ Conc. ~ bmi (lmer)					-0.729 (±0.704), p-value: 0.31789

Succinic acid

Equation	Age	Sex (male)	Voriconazole dose	Fasting time at T1	BMI
Δ Conc. ~ age + sex + Wmg_VRC + fastingtime_T1 + bmi (lmer)	-0.095 (±0.127), p-value: 0.46837	-2.331 (±2.377), p-value: 0.34381	0.000 (±0.004), p-value: 0.99	0.276 (±0.609), p-value: 0.65486	0.087 (±0.414), p-value: 0.83749
Δ Conc. ~ age + sex + Wmg_VRC + fastingtime_T1 (lmer)	-0.093 (±0.122), p-value: 0.45674	-2.260 (±2.272), p-value: 0.33612	0.000 (±0.004), p-value: 0.962	0.297 (±0.596), p-value: 0.62323	
Δ Conc. ~ age + sex + Wmg_VRC + bmi (lmer)	-0.089 (±0.125), p-value: 0.49194	-2.626 (±2.272), p-value: 0.27015	-0.001 (±0.003), p-value: 0.83344		0.117 (±0.406), p-value: 0.77879
Δ Conc. ~ age + sex + Wmg_VRC (lmer)	-0.086 (±0.120), p-value: 0.48629	-2.559 (±2.180), p-value: 0.26132	-0.001 (±0.003), p-value: 0.85423		
Δ Conc. ~ age + sex + fastingtime_T1 + bmi (lmer)	-0.095 (±0.126), p-value: 0.46658	-2.335 (±2.330), p-value: 0.33455		0.275 (±0.533), p-value: 0.61179	0.087 (±0.409), p-value: 0.8348
Δ Conc. ~ age + sex + fastingtime_T1 (lmer)	-0.094 (±0.122), p-value: 0.45462	-2.278 (±2.231), p-value: 0.32461		0.286 (±0.526), p-value: 0.59222	
Δ Conc. ~ Wmg_VRC (lmer)			-0.000 (±0.003), p-value: 0.95855		
Δ Conc. ~ fastingtime_T1 (lmer)				0.345 (±0.509), p-value: 0.50414	
Δ Conc. ~ bmi (lmer)					0.048 (±0.399), p-value: 0.90622

Acetone

Equation	Age	Sex (male)	Voriconazole dose	Fasting time at T1	BMI
Δ Conc. ~ age + sex + Wmg_VRC + fastingtime_T1 + bmi (lmer)	-0.041 (±0.072), p-value: 0.57875	-1.700 (±1.444), p-value: 0.26022	-0.001 (±0.005), p-value: 0.8103	-0.433 (±0.608), p-value: 0.48302	0.338 (±0.240), p-value: 0.18457
Δ Conc. ~ age + sex + Wmg_VRC + fastingtime_T1 (lmer)	-0.039 (±0.077), p-value: 0.61995	-1.191 (±1.488), p-value: 0.43622	0.001 (±0.004), p-value: 0.87873	-0.154 (±0.599), p-value: 0.7992	
Δ Conc. ~ age + sex + Wmg_VRC + bmi (lmer)	-0.050 (±0.073), p-value: 0.50447	-1.226 (±1.324), p-value: 0.37263	0.000 (±0.004), p-value: 0.93977		0.289 (±0.237), p-value: 0.24487
Δ Conc. ~ age + sex + Wmg_VRC (lmer)	-0.043 (±0.075), p-value: 0.57558	-1.037 (±1.347), p-value: 0.45493	0.001 (±0.004), p-value: 0.79303		
Δ Conc. ~ age + sex + fastingtime_T1 + bmi (lmer)	-0.041 (±0.073), p-value: 0.58666	-1.576 (±1.385), p-value: 0.2753		-0.357 (±0.557), p-value: 0.52685	0.323 (±0.234), p-value: 0.1939

Equation	Age	Sex (male)	Voriconazole dose	Fasting time at T1	BMI
Δ Conc. ~ age + sex + fastingtime_T1 (lmer)	-0.040 (±0.076), p-value: 0.60574	-1.246 (±1.422), p-value: 0.39539		-0.182 (±0.555), p-value: 0.7451	
Δ Conc. ~ Wmg_VRC (lmer)			0.002 (±0.004), p-value: 0.65003		
Δ Conc. ~ fastingtime_T1 (lmer)				-0.084 (±0.501), p-value: 0.86798	
Δ Conc. ~ bmi (lmer)					0.260 (±0.226), p-value: 0.27022

Caffeine

Equation	Age	Sex (male)	Voriconazole dose	Fasting time at T1	BMI
Δ Conc. ~ age + sex + Wmg_VRC + fastingtime_T1 + bmi (lmer)	-0.891 (±1.054), p-value: 0.41344	-34.781 (±20.840), p-value: 0.11656	0.055 (±0.062), p-value: 0.38101	-0.551 (±8.422), p-value: 0.94838	3.478 (±3.481), p-value: 0.33601
Δ Conc. ~ age + sex + Wmg_VRC + fastingtime_T1 (lmer)	-0.860 (±1.054), p-value: 0.42888	-30.359 (±20.377), p-value: 0.1571	0.069 (±0.060), p-value: 0.26518	1.656 (±8.127), p-value: 0.84009	
Δ Conc. ~ age + sex + Wmg_VRC + bmi (lmer)	-0.904 (±1.016), p-value: 0.39058	-34.194 (±18.414), p-value: 0.0872	0.057 (±0.056), p-value: 0.32449		3.418 (±3.289), p-value: 0.31852
Δ Conc. ~ age + sex + Wmg_VRC (lmer)	-0.819 (±1.016), p-value: 0.43435	-32.009 (±18.352), p-value: 0.10413	0.065 (±0.056), p-value: 0.25873		
Δ Conc. ~ age + sex + fastingtime_T1 + bmi (lmer)	-0.962 (±1.047), p-value: 0.37575	-40.030 (±19.923), p-value: 0.06476		-3.394 (±7.771), p-value: 0.66573	4.165 (±3.382), p-value: 0.24123
Δ Conc. ~ age + sex + fastingtime_T1 (lmer)	-0.942 (±1.065), p-value: 0.39217	-36.133 (±19.999), p-value: 0.09177		-1.548 (±7.688), p-value: 0.84189	
Δ Conc. ~ Wmg_VRC (lmer)			0.082 (±0.055), p-value: 0.15303		
Δ Conc. ~ fastingtime_T1 (lmer)				1.239 (±7.366), p-value: 0.86754	
Δ Conc. ~ bmi (lmer)					3.009 (±3.595), p-value: 0.41654
